# Microbial community characterization of multi-crop growouts in the XROOTS aeroponic–hydroponic system on the International Space Station

**DOI:** 10.3389/frmbi.2026.1779816

**Published:** 2026-06-15

**Authors:** Christina L. M. Khodadad, Cory J. Spern, Mary E. Hummerick, Jennifer L. Gooden, Cristiana J. Morales, Raymond M. Wheeler, Orlando Melendez, Robert Morrow, John Wetzel, Ye Zhang

**Affiliations:** 1 Life Sciences Laboratory, LASSO II, Noetic Strategies, Inc., Kennedy Space Center, FL, United States; 2 Amentum, Inc., LASSO I, Kennedy Space Center, FL, United States; 3 KBRWyle, Kennedy Space Center, FL, United States; 4 Bennett Aerospace, Inc., LASSO II, Kennedy Space Center, FL, United States; 5 Utilization and Life Sciences Office, NASA Kennedy Space Center, Kennedy Space Center, FL, United States; 6 Environmental Systems, Sierra Space, Inc., Madison, WI, United States

**Keywords:** aeroponics-hydroponics, International Space Station (ISS), microbiome, spaceflight, Veggie, XROOTS

## Abstract

Plant growth systems tested on the International Space Station (ISS) are small-area growth units that mostly use solid media. With NASA’s plan to send astronauts on long-duration exploration missions, there is a need to produce larger amounts of fresh food with limited upmass and resources. The eXposed Root On-Orbit Test System (XROOTS) is an aeroponic–hydroponic nutrient delivery system designed for exploration missions and was tested on the ISS. Post-harvest samples were returned for microbiological analyses of the plant leaves, roots, and fruit from lettuce, mizuna mustard, wheat, radish, tomato, and pea plants grown in the XROOTS. The microbiological food safety of crops was evaluated through culture-based microbial enumeration and identification. The microbial communities were compared between different plants and plant tissues by sequencing the prokaryotic V4 variable region of the 16S ribosomal RNA (rRNA) gene amplicons and fungal internal transcribed spacer (ITS) region. The microbial counts from the root module surface samples demonstrated a reduction after cleansing. The bacterial counts in the nutrient solution ranged from 65 to 3,800 CFU/ml. The bacterial counts in the distal leaf sections were lower than those in the leaf proximal, wick, and roots in all plant samples. The tomato fruit and the pea pod samples had the lowest average counts. The microbial counts from the leaves and wicks harvested from XROOTS were similar to the ranges found on previous Veggie (Vegetable Production System)-grown leafy greens. All screening tests for potential foodborne pathogenic bacteria were negative. Sequencing analyses showed that diversity was low in the leaves and higher in the roots, and the microbial community was more diversified in the XROOTS samples compared with previous Veggie experiments. *Pseudomonas* had the highest relative abundance in the majority of samples. Although some microbes were shared in the majority of plant tissues, unique microbes were identified for each plant type grown in XROOTS and when compared with previous Veggie demonstrations. Microbial surveys of ISS-grown plants and the associated hardware provide valuable data that can reveal potential challenges in deep-space crop production operations and ensure the quality of crops intended for crew consumption.

## 1 Introduction

Current plant growth chambers on the International Space Station (ISS) have a relatively small growth area, primarily supporting passive, solid media-based systems operating under unique spaceflight conditions. These passive solid media-based systems normally require a frequent watering schedule and experience anomalies with water and nutrient delivery and may require additional crew attention. As these systems are small, they provide a limited amount of food supplements for crew consumption and need to be up-scaled to provide sufficient nutritional benefits for the astronaut crew.

The National Aeronautics and Space Administration (NASA) plans to send astronauts to the moon and Mars with missions lasting from weeks to years. These exploration missions will require the ability to produce edible crops consistently and in sufficient quantity to feed the crew as resupply missions may not be available. Scaling up crop production will require additional investigations into substrate-free systems, which may substantially reduce the cargo mass transported to space destinations and simplify on-orbit, continuous plant production operations. Substrate-free systems include aeroponic and hydroponic systems that partially or completely submerge plant roots. An aeroponic system exposes the plant root system, making it available to receive water droplets or misting via an aerosol or other method *versus* the hydroponic system that partially or completely submerges the plant roots in nutrient solution. Aeroponic cultivation can provide up to 90% water conservation over more conventional watering methods (Eldredge et al., 2020; Rajaseger et al., 2023; Resh, 2022), which needs to be further investigated in the space environment with altered gravity.

**Figure 1:**
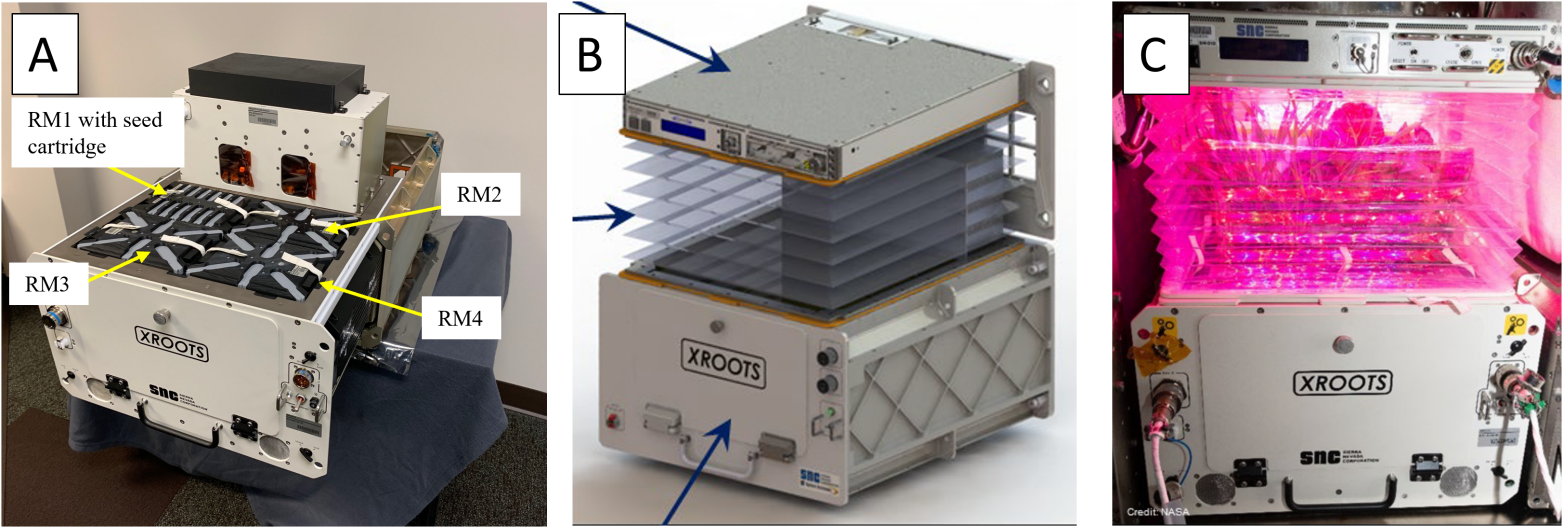
**(A)** The eXposed Root On-Orbit Test System (XROOTS) payload showing the four root modules (RMs) with seed cartridge. **(B)** XROOTS housed within the Veggie unit. *Arrows* (*top* to *bottom*) indicate the Veggie lighting canopy, the bellows, and the XROOTS hardware. **(C)** XROOTS with the Veggie light canopy activated on the International Space Station (ISS).

Substrate-free systems on Earth can sustain productivity and may be easier to maintain in spaceflight. In addition, they allow easy recovery of root biomass, which expands fundamental research capabilities with plants in space. To explore whether a substrate-free system is feasible for growing plants in the spaceflight environment, a aeroponic–hydroponic payload called “eXposed Root On-Orbit Test System” (XROOTS) was developed by Sierra Space, Inc., and was investigated on the ISS. The technology demonstration (tech demo) mission of XROOTS was completed on the ISS in 2022 (Wetzel et al., 2023). The XROOTS is a middeck locker equivalent (MLE) payload containing four quadrants and utilizing an aeroponic–hydroponic system within each quadrant to grow plants without substrate or other particulate solid media (**Figure 1**).

During the tech demo, the XROOTS hardware was integrated in the Vegetable Production System (Veggie) on ISS utilizing the lighting platform (Massa et al., 2017) (**Figures 1B, C**). Each quadrant consisted of one root module (RM) assembly, which includes a root chamber that provides water/nutrient delivery, the RM enclosure that provides the mounting capability, and the root/shoot interface that separates the root and shoot zones and serves as a platform for holding the seed cartridges (Wetzel et al., 2023) (**Figure 1A**). The nutrient delivery system was meant to be either an aeroponic spray or an ebb/flow hydroponic system or a combination with a variable delivery rate and duration with the intention of reducing crew time. Due to a malfunctioning valve, the hydroponic system for nutrient recovery was employed with astronaut assistance every third day. The RMs and seed cartridges (**Figure 1A**) were designed for multi-crop growouts (growth time from planting to harvest) to investigate how each crop adjusts or grows under different nutrient delivery cycles. Multi-crop growouts, similar to a previous Veggie technical demonstration completed in 2017 (Hummerick et al., 2021), provide an opportunity to offer diet variability and to study crop-to-crop interactions. The primary goal of the XROOTS ISS tech demo was to determine whether the XROOTS hardware performs well in microgravity through crop plant growout tests (including food safety evaluation). Furthermore, there are three hypothesis-driven scientific goals to determine: 1) whether crop plants grow successfully in XROOTS in microgravity; 2) whether the microbial communities in plants grown in the aeroponic–hydroponic system of XROOTS are comparable to those grown in solid substrate systems; and 3) whether the differences in the microbial communities are tissue type-, plant type-, location-, or process-dependent. Due to the nature of the tech demo, ground controls were not conducted. However, we compared the data with ISS historical datasets.

The objectives for the XROOTS tech demo were to investigate and evaluate a substrate free nutrient delivery system and different planting configurations in microgravity conditions. Four growouts of numerous types of crop plants were grown to either test germination or to maturity. Selected plants from three growouts were harvested and frozen in the Minus Eighty-Degree Laboratory Freezer for ISS (MELFI) for subsequent sample analyses after return to Earth. This mission provided the first ever test operating an aeroponic–hydroponic multi-crop system on orbit to grow crop plants. Upon return, the samples were analyzed for potential foodborne pathogens and to identify microbial communities in plant tissue samples, on hardware surfaces, and in nutrient solution samples. These data provide evidence of crop suitability and food safety for crew consumption of hydroponically grown crops in space.

## 2 Materials and methods

### 2.1 Planting on the ISS

The XROOTS tech demo payload was launched to the ISS on February 19, 2022, on NG-17 (Northrup Grumman) and installed in the Veggie unit in April 2022. Seed cartridges containing pre-planted unsanitized seeds from multiple crop types were inserted on the root/shoot interface by an ISS crew member before each growout. The XROOTS series of growouts were initiated in June 2022. Nutrient solution was created from ISS potable water with two sets of nutrient capsules: one set containing part A and another set containing part B of a concentrated Jacks two-part granular hydroponic mix (JR Peters Inc., Allentown, PA, USA). Part A was a Jacks Professional Nutrients 5–12–26 water-soluble fertilizer mix, while part B was Jacks Nutrients 15–0–0 (Wetzel et al., 2023). The nutrient solution was applied during the growout, with minor adjustments based on lessons learned from each previous growout. Growout 1 was used to refine the nutrient delivery and fluid recovery protocols (Wetzel et al., 2023). No plants were recovered from this first growout. Growouts 2–4 resulted in plants growing to mature stages (**Table 1**). Mature plant tissues were harvested from mizuna mustard, cherry belle radish, and little gem lettuce plants from growout 2; dwarf wheat and red romaine cv. ‘Outredgeous’ lettuce plants from growout 3; and the dwarf pea and tomato plants from growouts 3 and 4. The growth of dwarf pea and tomato plants was initiated in growout 3 in RM3 and RM4, respectively. Tomato plants were thinned to one plant and carried out to growout 4 for additional growth time. The pea plant was transplanted to RM4 at the start of growout 4 (Wetzel et al., 2023). The dwarf pea and tomato plants were grown to 84 days, when fruits developed.

**Table 1:** The eXposed Root On-Orbit Test System (XROOTS) growouts 2 through 4 harvested for post-flight analyses.

Growout	Root module (RM)	Plants	No. of plants analyzed	Dates
Growout 2	RM1	Mizuna mustard	1	Planted June 27, 2022, harvested July 27, 2022 (30 DAP)
	RM1	Cherry belle radish	1	Harvested July 27, 2022 (30 DAP)
	RM2	Little gem lettuce	1	
	RM3	Cherry belle radish	1	
	RM4	Cherry belle radish	1	
Growout 3	RM1	Dwarf wheat	3	Planted August 8, 2022, harvested September 2022 (39 DAP)
	RM2	Red romaine lettuce cv. Outredgeous	2	Harvested September 2022 (39 DAP)
	RM3	Earligreen dwarf pea (planted)	–	
	RM4	Micro Tina tomato (planted)	–	
Growout 4	RM4	Micro Tina tomato	1^a^	Planted August 8, 2022, harvested October 31, 2022 (84 DAP)
	RM4	Earligreen dwarf pea	1	Harvested October 31, 2022 (84 DAP)

The growth of tomato and pea plants was initiated in growout 3 and extended through growout 4 for longer development time until fruiting occurred.

*DAP*, days after planting.

^a^ Three subsamples of tomato plant leaves were taken from the same plant.

Each plant was harvested as two separate parts: the leaf tissues located above the seed cartridge (**Figure 1A**) and the root tissue from the lower compartment/RM (some with seed cartridges). For the pea and tomato plants, the pea pod and tomato fruits were harvested in separate bags. Leaves were collected from the top section of the plant. Each part collected was wrapped with sanitized aluminum foil, and all the samples were frozen and stored at −80°C in MELFI on the ISS until returned to the Kennedy Space Center (KSC) for analysis. Nutrient fluid samples and swabs from the interior corners and side surfaces of the RMs were also collected before and after growouts 2–4. These samples were frozen and stored at −80°C in MELFI on the ISS. After the sample return, all the samples were stored in a −80°C freezer at KSC until processed.

### 2.2 Microbiological sample processing

**Figure 2:**
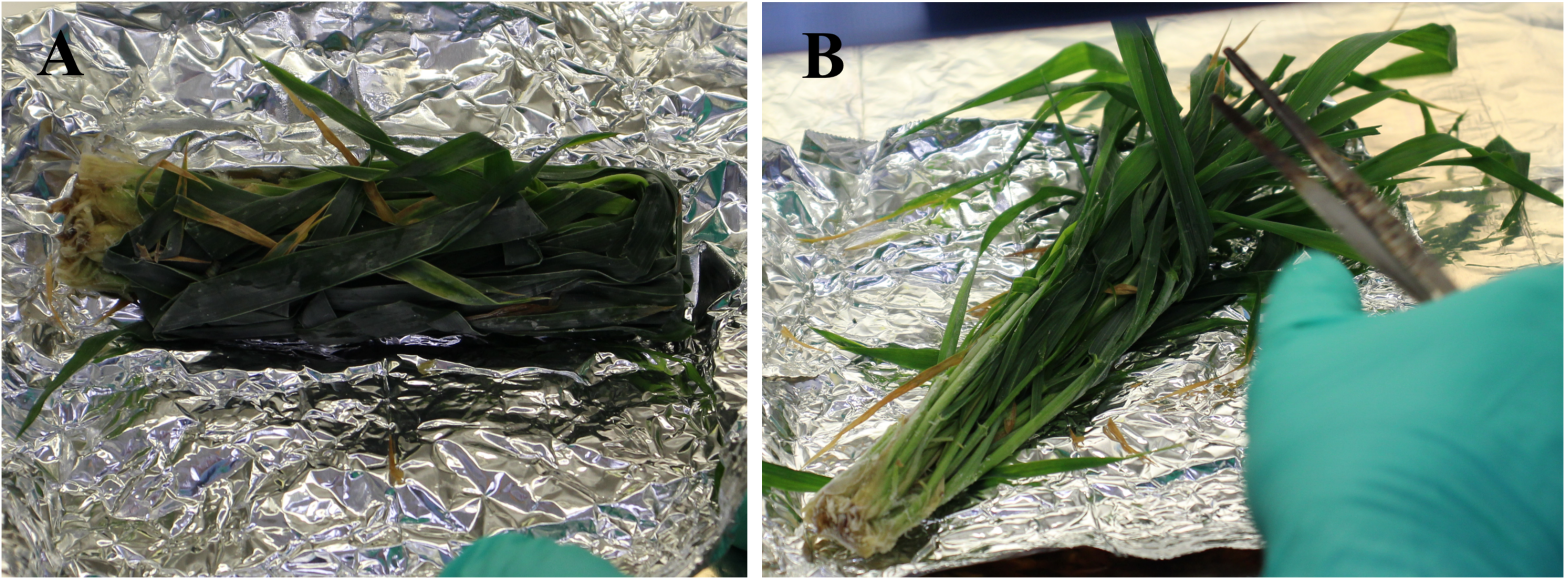
**(A)** Dwarf wheat leaf sample returned from the International Space Station (ISS) eXposed Root On-Orbit Test System (XROOTS) technology demonstration folded back, causing potential direct contact between the leaf sections. **(B)** The wheat plant extended for sampling.

Harvested samples were removed one at a time from the −80°C freezer and processed to minimize sample degradation during thawing. Slight thawing was necessary to cut the samples according to tissue location. The leaf samples were divided into a top and a bottom section at 2–3 in. from the wick. The root samples were also divided into sections closer to the wick (top) and the lower part of the root (bottom). Tomato fruit, pea pod, radish storage root, and the wick material retrieved from the root samples were collected separately and processed similarly to the leaf and root sections described below. Some of the larger plant samples were folded in the packaging so that it was possible that the top part of the plant tissue may have been in contact with the bottom section of the tissue (**Figure 2**). Each dissected tissue part was placed into a pre-weighed 50-ml centrifuge tube with sterile phosphate-buffered saline (PBS) and approximately 4 ml of 3- to 4-mm sterile glass beads, weighed, and then shaken for two cycles at 5 m/s for 30 s each on the Omni Bead Ruptor (OMNI, Kennesaw, GA, USA).

The sterile buffer containing swabs was allowed to thaw and was vortexed at high speed for 30 s before plating for microbiological and genomic analyses. Nutrient solution samples were thawed and processed as the swab samples.

### 2.3 Microbiological analysis

#### 2.3.1 Total counts and identification

All sample buffers from plant tissue, wicks, and swabs, as well as the nutrient solution samples, were serially diluted with sterile PBS and plated in duplicate onto both trypticase soy agar (TSA) for bacteria and inhibitory mold agar (IMA) for fungi. Plates were incubated at 30°C for 48 h for bacteria and 72–120 h for fungi, and the colonies were enumerated and presented as colony forming units (CFU). Individual colony phenotypes were selected from each sample and isolated on the respective agars. Bacterial colonies were identified using the Biolog Micro ID System (Biolog, Hayward, CA, USA). The bacterial colonies unidentified by Biolog and all fungal phenotypes for each sample were isolated for identification with the MicroSeq 16S rDNA or the MicroSeq D2 LSA rDNA using the ABI 3500 Genetic Analyzer Sequencing System (Thermo Fisher, Waltham, MA, USA). DNA was isolated using either the PrepMan Reagent (Thermo Fisher, Waltham, MA, USA) or the Qiagen Powerlyzer Soil Kits (Qiagen Inc., Carlsbad, CA, USA). The MicroSeq samples were analyzed using the instrument’s MicroSeq ID Microbial Identification Software v3.1-2019 (Thermo Fisher, Waltham, MA, USA). Sequences not identified by the MicroSeq library were analyzed with the NCBI Basic Local Alignment Search Tool (BLAST).

#### 2.3.2 Food safety screening

Petri films were used to identify and enumerate *Escherichia coli*/coliform and *Staphylococcus aureus* for microbial food safety screening (3M, St. Paul, MN, USA). Petri films were incubated at 35°C for 24 h according to the manufacturer’s guidelines, and any colonies present were enumerated, isolated, and identified using Biolog GEN III plates. To screen for *Salmonella*, 1 ml of sample buffer was added to 5 ml of buffered peptone water (BPW) and incubated at 35°C for 24 h. A 1-ml aliquot was then transferred into a 5-ml aliquot of Rappaport–Vassiliadis (RV) broth (Thermo Fisher, Waltham, MA, USA) and incubated for an additional 24 h at 35°C. The broth cultures were then streaked onto selective media for *Salmonella* and incubated at 35°C for 24–48 h according to the Food and Drug Administration (FDA) Bacteriological Analytical Manual.

### 2.4 Molecular analysis

#### 2.4.1 DNA isolation

The remaining solution of each processed sample described in *Section 2.2* was filtered using a 70-μm cell strainer to remove debris. The filtered liquid extract from each individual sample (plant tissue, wick material, or swab sample) was aliquoted into microfuge tubes, centrifuged at 13,000 × *g* for 3 min, and the pellets were collected. Each nutrient solution and swab sample was placed in a 1.5-ml microfuge tube and then centrifuged as described above to collect the cell pellet for analysis.

DNA was isolated from the cell pellet from each sample using the Qiagen DNEasy Microbial Cell DNA Isolation Kit following the manufacturer’s protocol (Qiagen Inc., Carlsbad, CA USA). An OMNI Bead Ruptor (OMNI, Inc. Kennesaw, GA, USA) was used for mechanical cell disruption for 1 min at 5 m/s. The isolated DNA samples were eluted in 30–50 μl of buffer to ensure adequate concentration for downstream protocols. DNA was quantified using the QUBIT double-stranded DNA assays (Invitrogen, Inc., Grand Island, NY, USA). Plant DNA present in the plant tissue samples was not removed prior to sequencing.

#### 2.4.2 Microbial community characterization using 16S rRNA gene and fungal ITS amplicon sequencing

Dilutions were made to sample DNA to acquire a concentration of 0.5 ng/μl for polymerase chain reaction (PCR). Amplification was completed in duplicate per sample using 1 ng of DNA in each 20 μl reaction and barcoded 16S ribosomal RNA (rRNA) gene primers as described in Khodadad et al. (2020). Each primer was barcoded with a unique 8-nucleotide sequence for demultiplexing after sequencing. Briefly, PCR was completed at 95°C for 5 min to activate the enzyme, followed by 30 cycles of 95°C for 1 min for denaturation, 58°C for 1 min for annealing, followed by 72°C for extension. A final extension at 72°C for 10 min completed the amplification.

Post-PCR cleanup was completed using the Qiagen MinElute Kit (Qiagen Inc., Carlsbad, CA, USA). Elution was completed in 10–20 μl EB buffer (kit) and was sample-dependent based on the starting DNA concentration. The final concentration of the PCR amplicon product was determined using the QUBIT double-stranded High Sensitivity DNA Assay (Invitrogen, Inc., Grand Island, NY, USA). An equimolar concentration was determined from the sample concentrations, and dilutions were created using TRIS buffer. An equal volume of each amplicon/sample was pooled to create the initial library, followed by final library preparations according to the Illumina protocols in preparation for sequencing. Sequencing was completed on the Illumina MiSeq sequencer with a V2 500-cycle sequencing kit.

Fungal identification was completed using the ZYMO Quick-ITS Plus NGS Library Prep Kit with approximately 10 ng of DNA following the manufacturer’s protocol (Zymo Research, Irvine, CA, USA). Briefly, 10 ng of DNA isolated from each sample was barcoded with unique 10-nucleotide dual indices and a PCR reaction completed as optimized for this kit. The sequencing library was built with an equal volume of each sample according to the kit protocol, cleaned with a bead protocol, and quantified with the QUBIT 2.0 double-stranded High Sensitivity DNA assay. The final library preparation for sequencing was based on the Illumina library prep and the MiSeq V2 500-cycle sequencing protocols with a 10% Phi-X spike to increase diversity in the chemistry.

#### 2.4.3 Bioinformatics analyses

Venn diagram analysis for the bacterial and fungal communities was completed using Venny 2.1 (https://bioinfogp.cnb.csic.es/tools/venny/index.html), and each bacterium with greater than 10 identified amplicon sequencing reads was included in the analyses (Oliveros, 2015). Due to the sparsity of the ITS read abundances, no read cutoff was applied for fungi.

Chloroplast and mitochondrial DNA sequences from plant tissue were removed from each sample prior to analysis. Analysis of the 16S rRNA bacterial amplicon sequences was completed using QIIME 2 v.2023.2 through Conda v.23.3.1 (Bolyen et al., 2019; McMurdie and Holmes, 2014; Hong et al., 2022) and included the generation of amplicon sequence variants (ASVs). Taxa were classified using SILVA v.138.99 (Quast et al., 2013). Principal coordinate analysis (PCoA) was conducted using R version 4.1.2 (Quast et al., 2013; R Core Team, 2021) with the following packages: QIIME2R v.0.996, tidyverse v.2.0.0 (Wickham et al., 2019), and phyloseq v.1.36.0 (McMurdie and Holmes, 2013). Any plant type with only one plant returned from the ISS was not included in the statistical analyses. The ITS sequences were analyzed via Kraken 2 (Wood et al., 2019). A custom database was created with ITS-specific DNA sequences retrieved from the UNITE database (UNITE general FASTA release for Fungi 2) (Abarenkov et al., 2022) to map with Kraken 2. Bracken (Bayesian re-estimation of abundance with KrakEN) was used to calculate the abundance of species from the Kraken 2 output. Finally, reports generated from the Kraken 2 mapping and Bracken’s species abundance estimates were visualized using Pavian, a web application for exploring metagenomics classification results (Breitwieser and Salzberg, 2016). All read abundance reports were generated at the genus taxonomic level (or higher taxa dependent on the database). Reports were imported into an R environment for visualization and were filtered to retain only those genera whose sum of reads across all samples was ≥10. Percent relative abundance of the top 20 most abundant bacterial and fungal genera was presented through stacked bar charts using the packages ggplot2 v.3.5.1 (Wickham, 2016) and dplyr v.1.1.4 (Wickham et al., 2023).

## 3 Results

### 3.1 Culture-based microbial counts and identification

#### 3.1.1 Microbial counts on hardware surfaces and in nutrient solution

Microbial counts on the RM surfaces and the bellows of Veggie (harvest 4 only) are shown in **Table 2**. Bacterial counts on the RM channels were reduced after cleaning with ProSan sanitizing wipes (Microcide, Sterling Heights, MI, USA), assuming the samples were collected in the same approximate area following the instructions.

**Table 2:** Bacterial and fungal counts (CFU/swab) from surface samples, acquired from the root modules of the eXposed Root On-Orbit Test System (XROOTS) on ISS.

Swab location	Harvest 2				Harvest 3				Harvest 4			
	RM1 before	RM1 after	RM3 before	RM3 after	RM1 before	RM1 after	RM2 before	RM2 after	Bellow bottom	Bellow top	RM4 before	RM4 after
TSA	3.5 × 10^5^	1.0 × 10^5^	9.9 × 10^4^	4.4 × 10^4^	7.6 × 10^5^	3.6 × 10^4^	1.5 × 10^5^	2.0 × 10^4^	3.9 × 10^3^	8.7 × 10^3^	6.6 × 10^4^	4.8 × 10^4^
IMA	3.5 × 10^3^	2.2 × 10^3^	4.8 × 10^3^	3.0 × 10^3^	1.7 × 10^4^	1.9 × 10^3^	2.1 × 10^3^	1.8 × 10^3^	2.5 × 10^1^	3.3 × 10^2^	2.4 × 10^3^	2.4 × 10^3^

Root module (RM) samples were taken before and after sanitization with ProSan wipes. Trypticase soy agar (TSA) was used to detect bacteria, while inhibitory mold agar (IMA) was used to detect yeast and mold.

The top of the Veggie bellows showed higher counts than the bottom of the bellows for both bacterial and fungal counts. The highest counts were from RM1 in harvest 3 (**Table 2**). The bacterial counts in the nutrient solution samples ranged from 3,800 to 65 CFU/ml, while the fungal counts were 660 CFU/ml, with the lowest at 25 CFU/ml (**Table 3**). The highest counts were also found in harvest 3 samples.

**Table 3:** Colony forming units per milliliter detected in the nutrient solutions used in the eXposed Root On-Orbit Test System (XROOTS) growouts.

	Harvest 2 harvest	Harvest 3 planting	Harvest 3 harvest	Harvest 4 harvest
TSA	8.4 × 10^2^	3.8 × 10^3^	6.5 × 10^1^	2.1 × 10^2^
IMA	2.5 × 10^1^	6.6 × 10^2^	7.0 × 10^1^	2.5 × 10^1^

Trypticase soy agar (TSA) was used to detect bacteria, while inhibitory mold agar (IMA) was used to detect yeast and mold. Sampling for harvest 3 was taken at seed initiation and again at harvest.

#### 3.1.2 Bacterial and fungal counts on plant and wick materials

**Figure 3:**
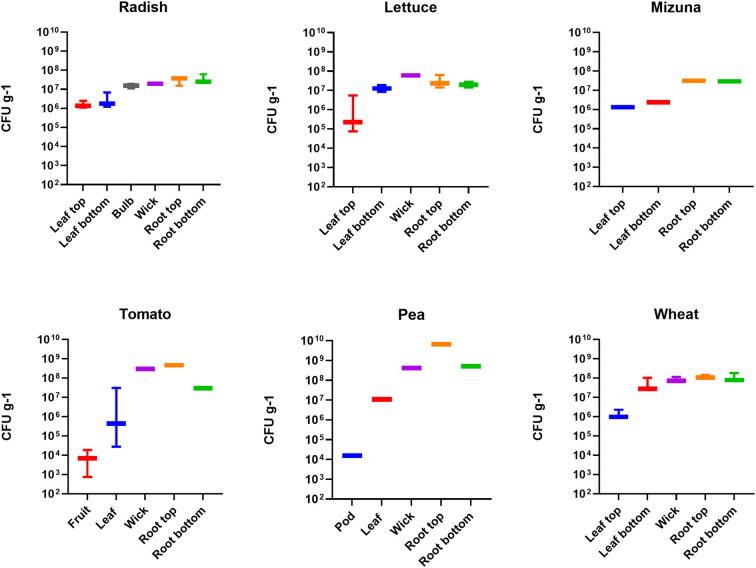
Bacterial counts [in colony forming units (CFU) per gram] in plant materials. *Vertical bars* indicate the maximum and minimum values where more than one sample was available for each sample type. The *bold bar* represents the median.

**Figure 4:**
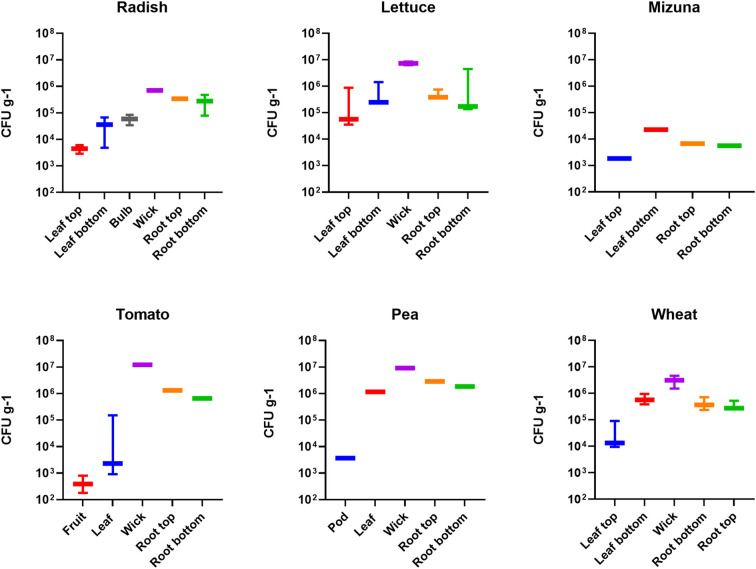
Fungal counts [in colony forming units (CFU) per gram] in plant materials. *Vertical bars* indicate the maximum and minimum values when more than one sample was available for that sample type. *Bold bar* represents the median.

All screening tests for potential foodborne pathogenic microbes were negative. The bacterial counts in the plant materials and wicks are shown in **Figure 3**. The bacterial counts in the leaf tops were lower than those on the leaf bottom, wick, and roots in all samples. The tomato fruit and the pea pod had the lowest average counts of approximately 10,000 CFU/g, while the highest counts were observed in the wick and roots. The microbial counts in the leaves harvested from XROOTS were within the range of counts found in the leaves from the previous VEG-01 and VEG-03 experiments (Khodadad et al., 2020; Hummerick et al., 2021). The fungal counts shown in **Figure 4** presented similar trends. The wick samples from XROOTS had the highest fungal counts, similar to those from previous Veggie samples (**Figure 4**).

Leaf and root samples from wheat, radish, and lettuce with at least three samples were analyzed further. Aerobic plate counts (APCs) and fungal counts (in CFU per gram) in wheat root *versus* radish and lettuce roots were greater (*p* = 0.023 and 0.016, respectively). There was no difference in the counts from the leaves across the three plant types (**Supplementary Table 1**).

When comparing the microbial load across sample types (leaves *vs*. roots *vs*. wicks) for each plant type, the roots had significantly higher APCs than the leaves in all plants compared (radish: *p* = 0.0027; lettuce: *p* = 0.0145; wheat: *p* = 0.0200). Only wheat had enough wick samples for comparison (*n* = 3). The APCs (in CFU per gram) of the wheat plant parts were not significantly different from those of the wick; however, the fungal counts were higher in the wick than in the leaves (*p* = 0.0154) and the roots (*p* = 0.0319) (**Supplementary Table 1**).

Individual colony types of bacteria and fungi were identified and are listed in **Supplementary Table 2**. Some of the isolates were found in all sample sets (i.e., the fungus *Fusarium*). In addition, *Chryseobacterium*, *Pseudomonas*, *Rhodotorula*, *Sporothrix*, *Exophiala*, and *Trichoderma* were found in the majority of sample sets. *Fusarium solani*, which is a common soil inhabitant and can cause root rot, was identified in plants, nutrient solution, and swabs from harvests 3 and 4. There may be some plant species-dependent patterns as well. For example, for the tomato and pea plant samples in the fourth harvest, the types of microbes were different, particularly for the leaf samples and the root bottom samples, even though both plants were grown in the same module. From the swab samples collected from the fourth harvest, it appeared that the microbial varieties located on the bellows were also different from those on the RM surfaces.

### 3.2 Diversity analyses

Sequencing of the microbial communities was completed using the V4 region of the 16S rRNA gene and the ITS region for fungi. Diversity of these communities was investigated within each sample (Shannon index) and between samples (Bray–Curtis).

#### 3.2.1 Alpha diversity

Alpha diversity was calculated using the Shannon index based on both the total number of taxa and their abundance (evenness of distribution). The data for the bacterial community indicated that leaf tissue had lower diversity than the wick and root samples, as expected (**Supplementary Table 3**). Comparison of the edible plant parts from harvest 2 showed that the radish bulbs had higher bacterial diversity than the lettuce and mizuna mustard leaves. Tomato fruit from harvest 4 had the lowest diversity and species number out of all samples, while the pea plant had varying indices (**Supplementary Table 3**). The nutrient solution and swabs had higher alpha diversity than the majority of leaf and root samples. The alpha diversity for the fungi was low (between 0.02 and 0.1) in the radish leaf and bulb, tomato fruit, and pea pod. Trends in diversity in the leaf tissues among different plants showed low diversity, particularly in the mizuna mustard and lettuce leaf samples. Among the plant types, wheat had a higher diversity and an increase in the number of genera within all leaf samples. Surface swabs yielded the highest alpha diversity followed by nutrient solution (**Supplementary Table 4**).

#### 3.2.2 Beta diversity

**Figure 5:**
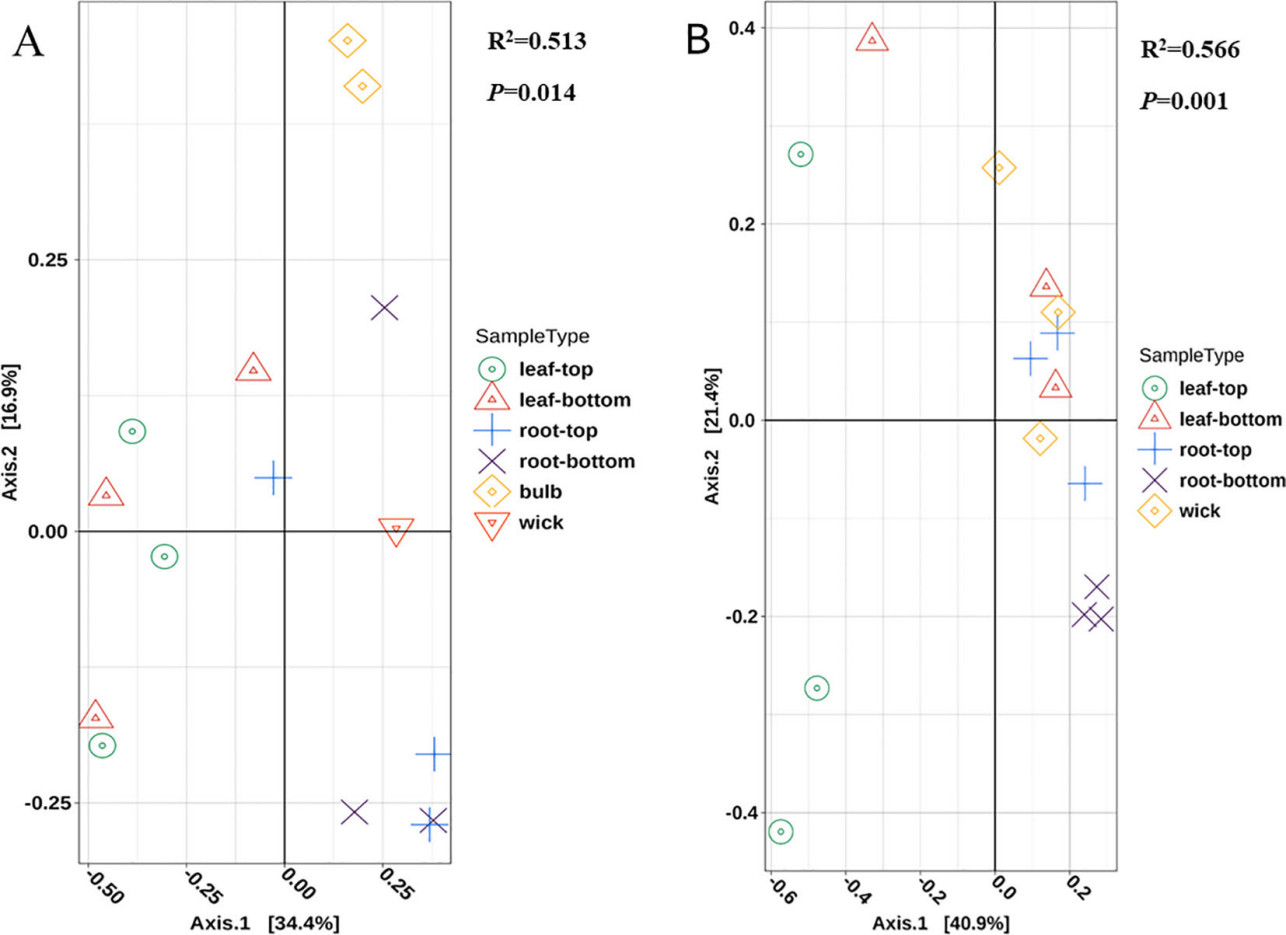
Ordination plots of the microbial community structure of radish **(A)** and wheat tissues **(B)** based on 16S rRNA gene amplicon sequencing. Ordination was performed using principal coordinate analysis (PCoA) based on the Bray–Curtis dissimilarity of the samples collected during growouts 2 and 3. The *R*^2^ value indicates the level of similarity/dissimilarity, and the *p*-value is the level of significance. *p* ≤ 0.05 is significant (*n* > 1).

Beta diversity is used to describe the diversity among samples. Using the Bray–Curtis (*R*^2^) statistic, between-sample diversity was determined for bacteria in each crop type with at least two samples returned. An *R*^2^ value closer to 1 indicates dissimilarity among samples, while an *R*^2^ value closer to 0 indicates similarity among the communities. The values of the axes combined may explain the influences of the factors that may affect the microbial community structure. Once identified, the *p*-value is calculated to indicate whether factors play a significant role (*p* < 0.05 is considered significant). The PCoA for the radish tissue was determined from three plants and revealed that 51.3% of the variation can be explained due to the sample type/tissue section. The remaining 48% was due to residual effects, which could be due to environmental factors, or other undetermined factors. The *R*^2^ value of 0.513 was significant at *p* = 0.014. There was no apparent clustering for the radish samples (**Figure 5A** and **Supplementary Table 5**).

The diversity within the wheat crop as displayed in **Figure 5B** showed some clustering in the root tissue. The combined axes explained approximately 62.3% and the *R*^2^ value was 0.566, indicating that 56.6% of the dissimilarity was due to sample type/tissue section. These results suggest that tissue type significantly contributed to the diversity more than other factors. However, due to the small sample size analyzed in this study, more investigations are needed to provide further evidence on this finding (**Supplementary Table 5**).

### 3.3 Community analysis and comparisons with previous Veggie technology demonstrations

The community constituents identified using the 16S rRNA gene or the ITS region were segregated by plant type, tissue type, and harvest to illustrate the similarities or the differences in the bacterial and fungal communities and were compared with data from previous Veggie demonstrations, i.e., VEG-03D, VEG-03E, and VEG-03F, using substrate-based root systems. Multiple comparisons to investigate changes in the microbial communities of plant tissues were presented using Venn diagrams.

#### 3.3.1 Bacterial community comparison among different crop types and tissue types

**Figure 6:**
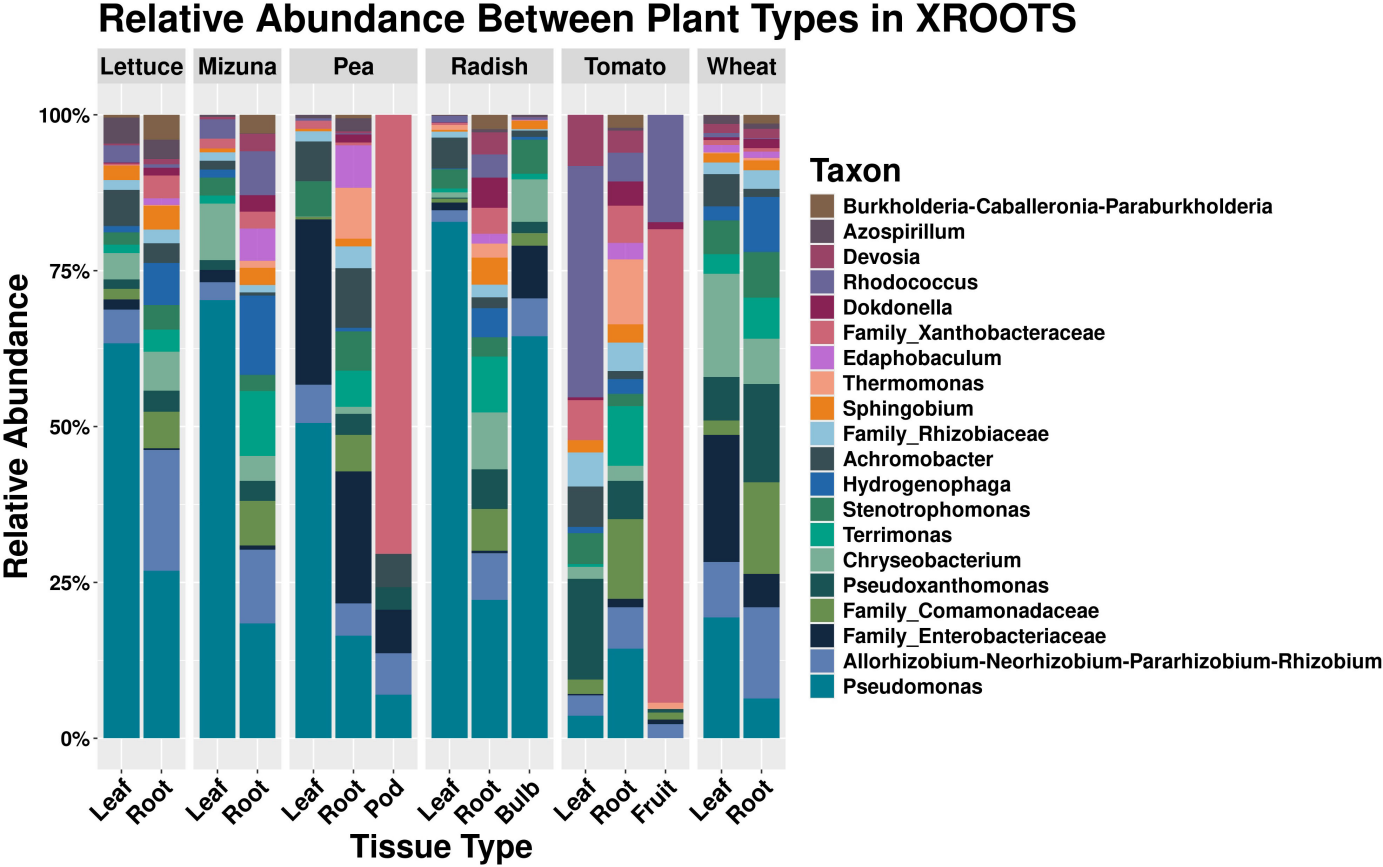
Microbial community composition of the plant tissues collected during all harvests. Stacked bar plots represent the average relative abundance of the 20 most abundant bacterial genera/families based on 16S rRNA gene amplicon sequencing and annotation using the SILVA v.138.99 database.

The top 20 abundant bacterial genera in the plant tissues (leaf, root, and fruit) were compared to preview the percent relative abundance (**Figure 6**). The microbial communities in the leaf and root appeared similar, with *Pseudomonas* present in high relative abundance particularly for leafy greens. The family Xanthobacteraceae, being the lowest taxonomic level detectable, showed higher abundance in the pea pod and tomato fruit compared with the leaf and root.

**Figure 7:**
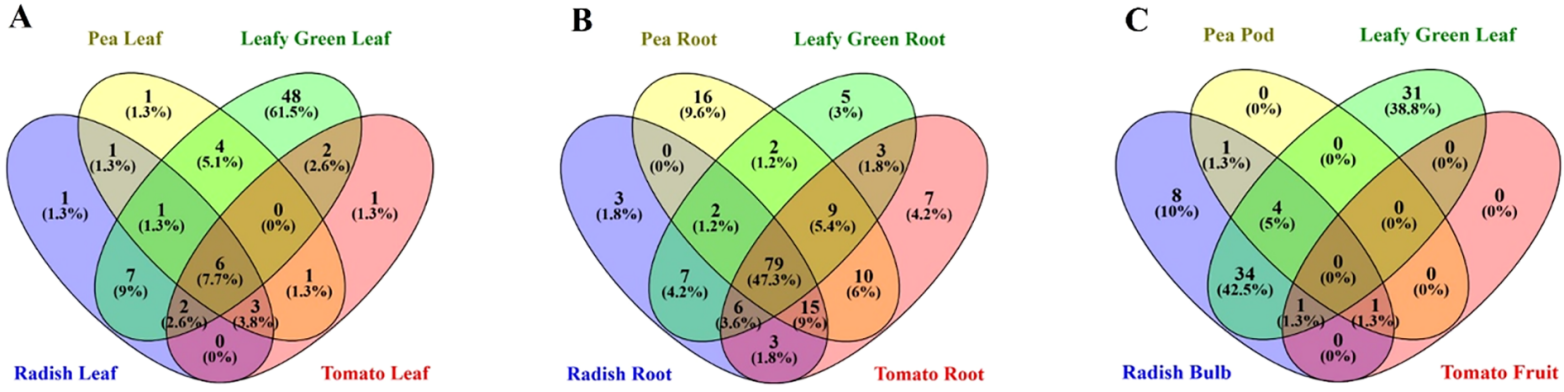
Comparison of the plant tissues harvested in the eXposed Root On-Orbit Test System (XROOTS) payload on the International Space Station (ISS) including leaf **(A)**, root **(B)**, and edible components **(C)**. Venn diagrams were completed with Venny 2.1 and show the number and percentage of the total shared or unique bacteria (*n* ≥ 1). https://bioinfogp.cnb.csic.es/tools/venny/index.html.

Comparisons with the Venn diagrams as displayed in **Figure 7** revealed that the bacterial communities in the leaf, root, and edible fruit had numerous unique variations. For example, comparisons of the leaf tissues from four crop types (leafy greens, tomato, radish, and pea) presented only six bacteria in common (*Pseudomonas*, *Achromobacter*, *Stenotrophomonas*, *Sphingobium*, *Rhizobium*, and *Rhodococcus*) (**Figure 7A**). Radish, pea, and tomato had very few unique bacteria, and the leafy greens (lettuce and mizuna mustard) had 48 bacterial genera (out of 70 total genera identified) not observed in the other plants. Among these bacteria were *Burkholderia*, *Mesorhizobium*, and *Sphingomonas.*

A comparison of the plant root tissue revealed a different outcome. The roots from these four crop types contained 79 genera (47.3%) in common, which included the six bacterial genera common to the plant leaves (**Figure 7B**). In addition to these six, *Bacillus*, *Hydrogenophaga*, *Stenotrophomonas*, *Sphingobium*, and *Pseudomonas* were abundant. The pea root had 16 unique genera, of which many were rarely detected in previously grown space crops. In addition, another 15 genera were detected in all but the leafy greens. Many of these genera, such as *Chitinophaga*, *Caulobacter*, and the group Comamonadaceae, were often detected in crops previously grown on ISS (**Figure 7B**).

The edible parts of the plants (i.e., pea pod, radish bulb, lettuce leaf, and tomato fruit) were also investigated to identify the microbial community and determine whether they are safe to eat (**Figure 7C**). Although these plants were grown in proximity (time and space), there appeared to be no bacteria common to all. Leafy greens possessed the highest number of unique microbes with 31 genera, with radish having eight unique bacterial genera. The leafy greens and the radish bulb shared 34 genera, including *Pseudomonas*, *Sphingobium*, *Rhizobium*, and *Burkholderia*. There were only two genera detected in the tomato fruit: *Rhodococcus* and *Xanthobacter* (**Figure 7C**).

#### 3.3.2 Comparison within each growout

**Figure 8:**
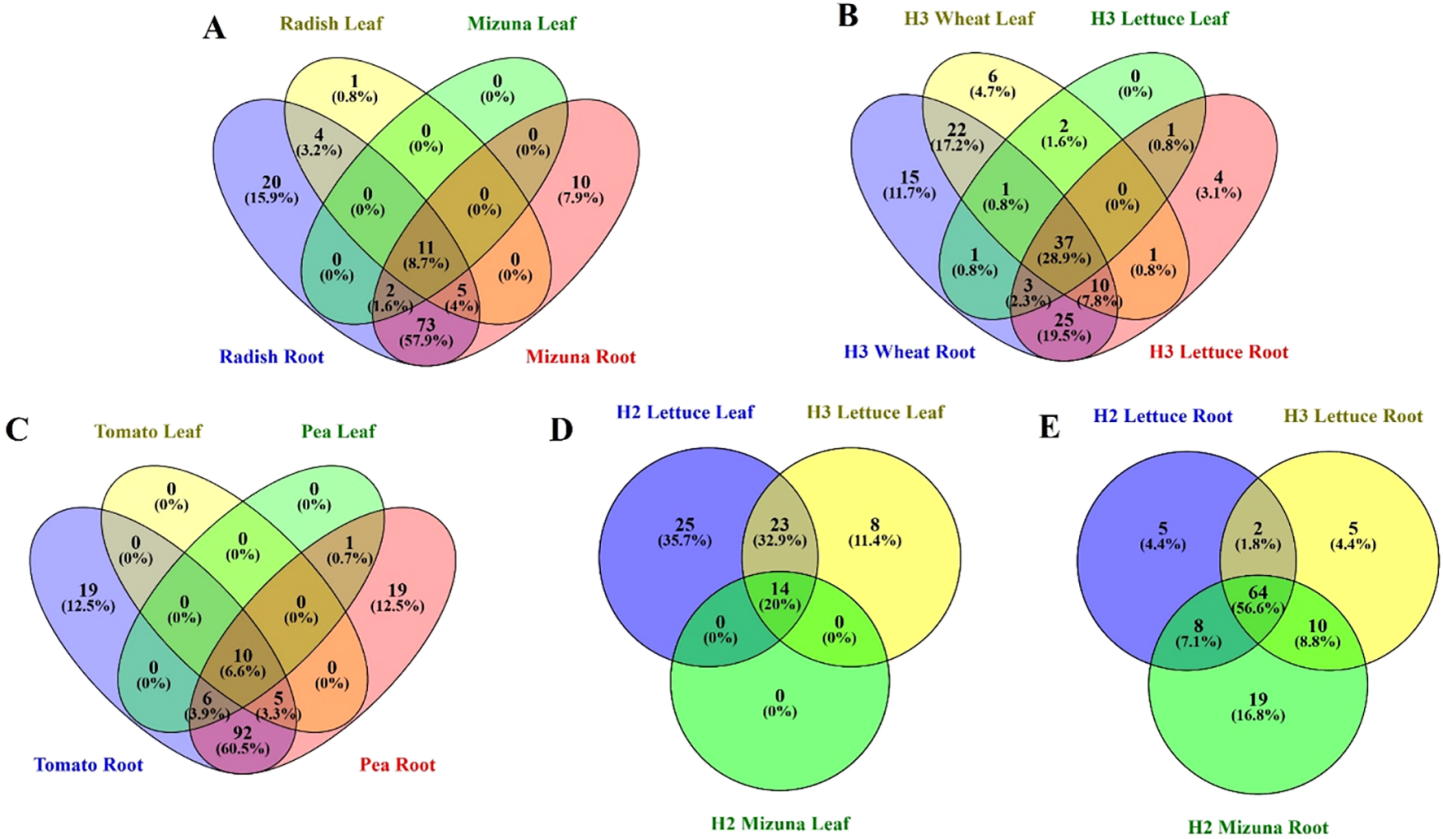
Comparison of the plant leaf and root tissue by harvest, including leafy greens from harvests 2 and 3, wheat from harvest 3, and tomato and pea harvested after growout 4 grown on the International Space Station (ISS) in the eXposed Root On-Orbit Test System (XROOTS) technology demonstration. The leaf and root tissues were compared within harvest 2 **(A)**, harvest 3 **(B)**, and harvest 4 **(C)**. Harvests 2 and 3 were compared between leaf **(D)** and root **(E)** tissues. Venn diagrams were completed using Venny 2.1 (*n* ≥ 1.0). https://bioinfogp.cnb.csic.es/tools/venny/index.html.

The XROOTS tech demo on the ISS was completed in separate growouts with separate harvests; therefore, a comparison of each harvest seemed warranted. Radish and mizuna mustard were grown and harvested during the second growout on ISS. In a comparison of the bacterial genera present in these plant types, only 11 genera were found to be common to all sample types. There were no more genera in common between radish and mizuna mustard leaves, but the roots shared 80 more genera (**Figure 8A**). Of these genera *Ralstonia*, *Cupriavidus*, and *Burkholderia* have previously been identified in ISS potable water (La Duc et al., 2003; Bruce et al., 2005; Ichijo et al., 2022). Growout and harvest 3 containing wheat and lettuce showed an increase in bacteria overall, with 37 shared genera among all the samples (**Figure 8B**). In addition, the roots of these two plants shared another 38 genera; however, the leaves shared only three more genera. The wheat roots had more unique genera (*n* = 15) than the lettuce (*n* = 4). Of these common taxa, we observed the same genera detected in other comparisons (*Rhizobium*, *Pseudomonas*, *Stenotrophomonas*, and *Burkholderia*, to name a few).

The pea plant and tomato plant that were initiated in growout 3 were continued into growout 4 as additional time to maturity and fruiting was required. The comparison of the leaf tissue between tomato and pea shown in **Figure 8C** indicated no common bacteria other than the 10 genera shared by all the samples. Neither tomato nor pea leaves presented any unique genera; however, the roots alone shared 92 genera. The 10 common genera shown in **Figure 8C** have also been identified in other comparisons, which included *Hyphomicrobium*. The roots from both the tomato and pea plants each presented 19 unique genera and shared an additional 11 genera with the tomato and/or pea leaf samples (**Figure 8C**).

There were three leafy greens grown and harvested in growouts 2 and 3 (two lettuce types and mizuna mustard). **Figures 8D, E** provide a comparison of these three leafy green leaves and roots to investigate potential microbial differences between the harvests. These leafy greens are often grown on ISS and consumed by the crew. The edible lettuce leaf from harvest 2 had the most unique genera (*n* = 25) compared with the lettuce crop grown in harvest 3, which had only eight unique genera. There were, however, 37 shared genera between the two lettuce crops. Mizuna mustard of harvest 2 had no unique genera, but there were 14 common genera with the two lettuce crops. Of these 14 common genera, we observed the same genera previously listed, including *Pseudomonas*, *Sphingobium*, *Rhizobia*, and *Stenotrophomonas.* In addition, *Bacillus*, *Devosia*, and *Methylobacterium* were common in the leaf tissue (**Figure 8D**). The root tissue of the lettuce and mizuna mustard crops again presented a different picture, with each lettuce crop having only five unique genera. Mizuna mustard root, however, had 19 unique genera. There were 64 genera common to all three harvested plants. The most abundant shared genera included *Burkholderia*, *Pseudomonas*, *Rhizobium*, and *Pseudoxanthomonas*, to name a few. This pattern is similar to that shown in **Figures 7A, B**, with more unique genera presenting in the leaf samples among different plants, but sharing common genera in the root samples.

#### 3.3.3 Nutrient solution samples

The nutrient solution samples collected from the three harvests shared 56 genera (**Supplementary Figure 1**). Harvests 3 and 4 had the highest number of unique genera, with 14 and 13, respectively, while harvest 2 had only six unique genera.

#### 3.3.4 Surface swabs

**Figure 9:**
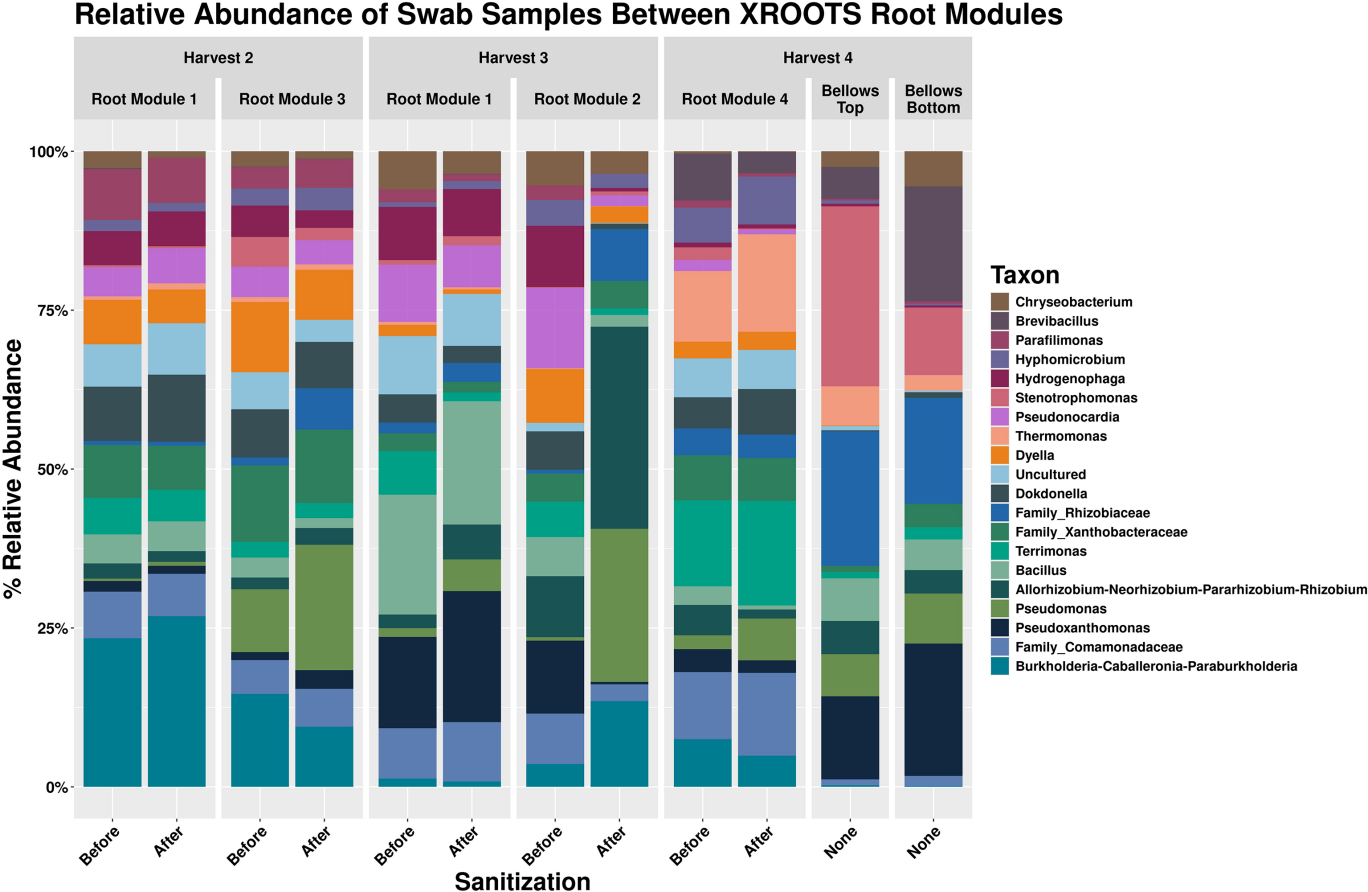
Stacked bar plots for the top 20 bacterial genera identified on swabs taken before and after cleaning with ProSan wipes for each root module. Taxa were identified using the 16S rRNA gene and the SILVA 138.99 database. Harvest 2 root module 1 (RM1) is mizuna mustard and RM3 is cherry belle radish. Harvest 3 RM1 is dwarf wheat and RM2 is red romaine lettuce. Harvest 4 RM4 is tomato and pea.

Although the colony count data showed reduced number in the RMs after cleaning (**Table 2**), swab samples of the RMs showed few differences in the relative abundance of the microbial communities before and after cleaning using ProSan wipes (**Figure 9**). The top genera with few differences in abundance included *Bacillus*, *Rhizobium/*Rhizobiaceae, *Chryseobacterium*, and *Pseudomonas*.

#### 3.3.5 Comparison between XROOTS and previous Veggie technology demonstrations

**Figure 10:**
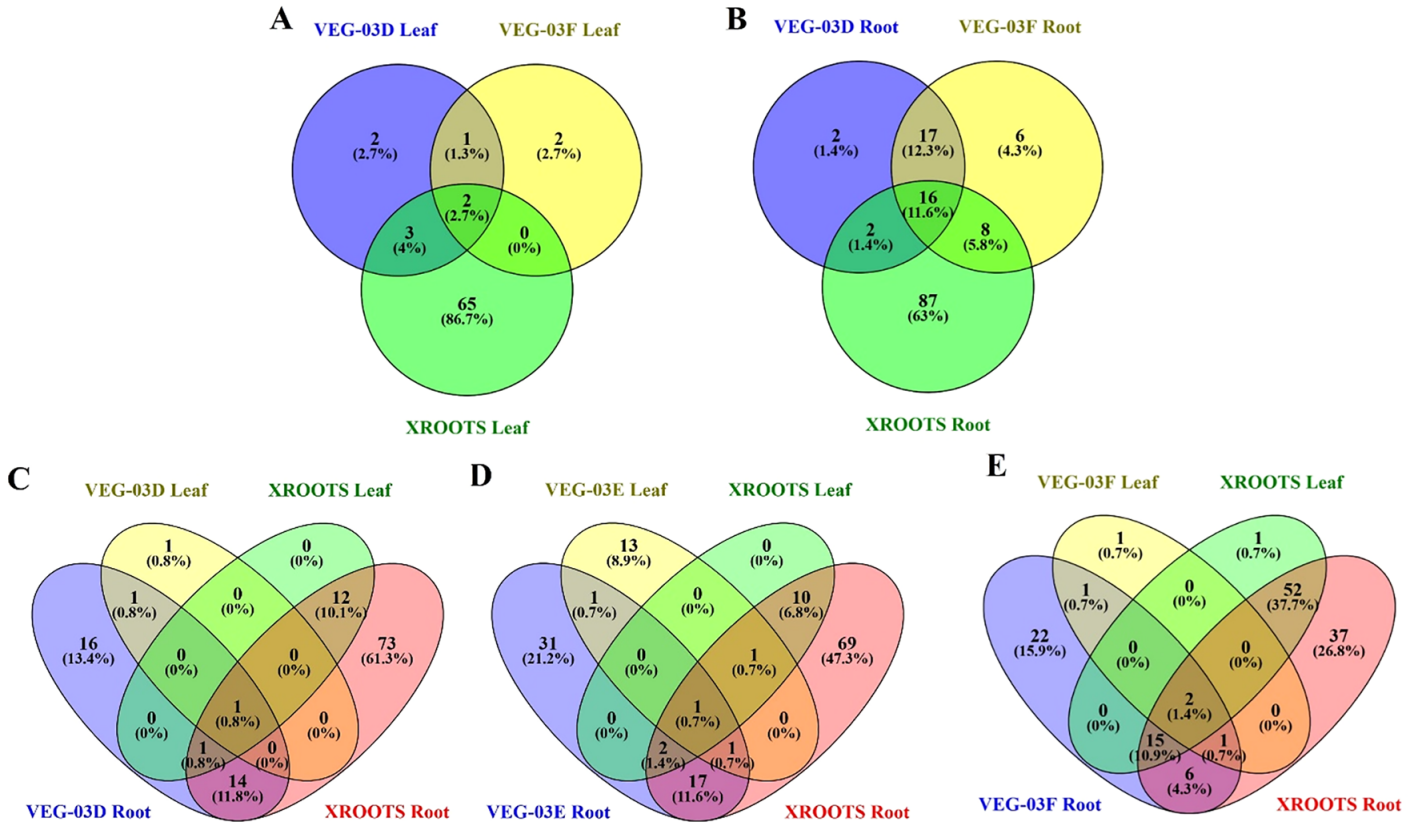
Comparison of the eXposed Root On-Orbit Test System (XROOTS) to the Veggie technology demonstrations of lettuce and mizuna mustard crops grown on the International Space Station (ISS). This includes lettuce leaf **(A)** and root **(B)** comparisons between XROOTS and VEG-03D and VEG-03F, as well as mizuna mustard leaf and root comparisons between XROOTS and VEG-03D **(C)** and VEG-03E **(D)**. A direct comparison of the lettuce leaf and root tissues between VEG-03F and XROOTS is also provided **(E)**. The Venn diagrams were created using Venny 2.1 (*n* ≥ 1). https://bioinfogp.cnb.csic.es/tools/venny/index.html.

Similar multi-crop growouts have been grown on the ISS, such as the tech demos VEG-03D, VEG-03E, and VEG-03F. Comparison with the VEG-03D, VEG-03E, and VEG-03F data (Hummerick et al., 2021) showed that the XROOTS lettuce leaf tissue had a few genera (*n* = 2) shared with VEG-03D and/or VEG-03F (**Figure 10A**). These two genera were *Pseudomonas* and *Burkholderia*. XROOTS tissues were more diverse in bacterial community, with 65 unique genera.

The XROOTS lettuce root tissue followed a similar pattern, with over 80 unique genera and only 26 genera shared with the VEG-03D and VEG-03F lettuce roots (**Figure 10B**), including genera found in ISS potable water and *Rhizobium* normally found in root microbiomes. A separate comparison between XROOTS and VEG-03D confirmed that, in mizuna mustard, there were more unique genera in the leaf and root than the overall shared genera (**Figure 10C**).

The leafy green mizuna mustard comparison between XROOTS harvest 2 and VEG-03E leaf showed similar results, but with very few shared genera, while the roots had 17 common genera with a much greater number of unique bacteria (**Figure 10D**). The VEG-03F lettuce leaf and root were also compared with the XROOTS lettuce leaf and root plant tissues. The leaf tissues of VEG-03F and XROOTS both had one unique genus (**Figure 10E**). The VEG-03F roots had 22 unique genera, whereas the root tissue of XROOTS showed 37 unique genera, with only two genera common to all. The XROOTS plants also shared more genera between the leaf and root (*n* = 69) *versus* the VEG-03F leaf and root tissues, which shared only four (**Figure 10E**).

### 3.4 Fungal communities throughout the components and crops

#### 3.4.1 Plant tissues

Analysis of the ITS data identifying the fungi present in each sample was completed by comparing the relative abundance to show the top fungi identified using the UNITE 2022 database. The number of genera was variable between plant types, plant tissue types, and edible fruits (**Supplementary Figure 1** and **S2**). Comparison of the leaf tissues from pea, tomato, leafy greens (lettuce and mizuna mustard), and radish showed a few fungi (*n* = 5) common between the four crop types, while the root tissues (**Supplementary Figure 1B**) shared 12. The edible plant portions shared only three fungal genera, with edible leafy greens showing more unique fungi. However, the non-edible tomato root presented 46 unique fungi and an additional 27 genera in the pea compared to 113 bacterial genera shared between pea and tomato roots. This is interesting as both the pea and tomato shared the same RM (RM4) during the fourth growout, indicating that bacterial and fungal harbering/distribution patterns are different and fungal distribution may be more plant type-specific. Additional comparisons between harvests indicated that harvest 3 shared the highest number of fungi with 14 genera in common (**Supplementary Figure 2**). The leafy greens between harvests 2 and 3 indicated six genera in common and 20 genera shared between the lettuce types. Each lettuce plant revealed unique genera, but mizuna mustard had no unique genera. A similar distribution pattern as identified in leaf samples was detected in plant roots.

#### 3.4.2 Nutrient solution samples

Fungi were present in all four nutrient solution samples to varying abundances, and the most abundant fungi were different in each sample (**Supplementary Figure 4**). The most abundant fungi in harvest 2 was *Fusarium* or *Sporothrix* and was similar to the post-harvest 3 sample, which had an increase in the abundance of *Fusarium.* The growout 3 sample taken at planting had a lower abundance of *Fusarium*, but a higher abundance of *Trichoderma* compared with the post-harvest 3 sample. Harvest 3 had the highest number of genera overall. The nutrient solution sample from harvest 4 indicated less diversity and lower abundance of the majority of the fungal genera detected at approximately 1% of the relative abundance. The dominant fungi in harvest 4 nutrient solution were *Blumeria* followed by *Innospora* (**Supplementary Figure 4**). These differences indicated that harvest 4 was more dissimilar than harvest 2 or 3.

#### 3.4.3 Surface swabs

The surface swab samples presented similar data to the plant tissue samples, with *Blumeria* higher in swabs from harvests 2 and 4 over harvest 3, ranging up to 80% of the total relative abundance (**Supplementary Figure 5**). Variable levels of *Fusarium* were identified in both harvest 3 (range, 10%–60%) and harvest 4 (range, 20%–70%). The same inverse abundance values were observed (higher *Fusarium*/lower *Blumeria*) (**Supplementary Figure 5**). The sanitization of the surfaces did appear to have a positive effect by lowering the relative abundance of some fungi, with *Blumeria* and *Fusarium* being exceptions in some cases.

#### 3.4.4 Comparison to Veggie technology demonstrations

A comparison of the XROOTS fungal communities to those identified in VEG-03D, VEG-03E, and VEG-03F was completed. Data for the lettuce plants from XROOTS and VEG-03D were compared, with a surprising outcome of only one fungus shared between the leaf and root tissues (**Supplementary Figure 6**). The XROOTS lettuce leaf tissue exhibited 29 unique genera not seen in the prior Veggie tech demos compared (**Supplementary Figure 6**). This was not unusual as the XROOTS seeds were not sanitized and there were at least eight additional Veggie tech demos and several other experiments that had utilized Veggie on ISS between VEG-03D, VEG-03E, and VEG-03F, and XROOTS. One of the fungal genera that had not been previously detected on ISS was *Blumeria.*

## 4 Discussion

With the need to enable crop production to achieve sustainable productivity for future long-duration exploration missions, feasible and effective methods of cultivation are needed. On Earth, soilless alternative methods of growing crops, including substrate-free methods, are being investigated to meet the increasing population growth. Substrate-free methods being developed include aeroponics, hydroponics, and a combination of both. Aeroponic methods use a water misting system to the roots to provide the nutrients required for plant growth (Garzón et al., 2023). This could be a more effective agricultural method when water is scarce, as experienced on the ISS and in deep space; however, further investigation is needed to validate the performance of an aeroponic system in an altered gravity environment. It has also been shown that hydroponic systems can increase productivity in a limited space while improving water use efficiency (Fathidarehnijeha et al., 2024; Edmonds et al., 2020). Water conservation is critical in spaceflight and for future moon and Mars exploration in order to maintain water availability, quality, and safety during missions.

Key to the success of a hydroponic system is the method of delivery of the nutrient solution to the root zone (Fathidarehnijeha et al., 2024). The XROOTS successfully used an aeroponic–hydroponic nutrient delivery system (Wetzel et al., 2023) in the microgravity environment with a recovery feature minimizing water usage. The use of these two methods in combination led to three successful, mixed-crop growouts of leafy greens, radish, wheat, and fruiting crops (tomato and pea) on the ISS.

### 4.1 Total microbial load and food safety

Growing mixed crops in a semi-closed to closed environment in spaceflight may introduce additional challenges, in particular concerning the plants’ microbial communities, which play a major role in plant success (Hummerick et al., 2021). Another concern is food safety: are the crops safe to consume? Here, we reported for the first time on the results of microbial communities and food safety associated with multi-crop plant growouts in an aeroponic–hydroponic system on the ISS.

Culture-based microbiological enumeration of bacteria and fungi indicated relatively consistent trends across plant types, with the leaf counts lower than those of the wick and root material. This is to be expected as the phyllosphere is generally low in available nutrients and the environmental conditions are not usually optimal for microbial growth (Lindow and Brandl, 2003). Determining the microbial load on the edible part of the plant provides data that characterize the quality and microbiological safety to the consumer. Edible leafy greens (lettuce or mustard leaves) grown in the field or hydroponically have been reported to have bacterial counts in the range of 1 × 10^4^–1 × 10^8^ CFU/g (Zhang et al., 2018; Bohaychuk et al., 2009; Wood et al., 2015) and fungal counts of 1 × 10^6^ CFU/g (Maffei et al., 2013). The lettuce and mizuna mustard leafy greens grown and harvested in XROOTS fall within these ranges, and when compared with other plant growth systems utilized on the ISS, the XROOTS plants were comparable (Bunchek et al., 2024; Khodadad et al., 2020; Hummerick et al., 2021). The ISS tomato fruits and radish bulbs also did not exceed the bacterial counts reported in the literature for tomato (10^3^–10^6^ CFU/g) and radish (10^4^–10^6^ CFU/g) (Klapec et al., 2016; Penteado et al., 2016; Bohaychuk et al., 2009). These data indicate that produce crops grown in XROOTS exhibit typical microbial loads for each type.

The standard screenings of the common food pathogens, *E. coli*, *Salmonella*, and *S. aureus*, were all negative. This result was later confirmed with culturable isolate identification and with the sequencing results using the 16S rRNA gene.

### 4.2 Microbial abundance among plant types and tissues

Microbial community dynamics are important to both plant growth and food safety. The plant microbial community is affected by the environmental conditions and by the nutrient solution supplied to the root zone of hydro-aeroponically grown crops (Sheridan et al., 2016; Andrade et al., 2025). Plant health and intercropping may also influence the community. Each of the three growouts was a multi-crop growout of two to three different plant types. Comparison of the microbial communities of the harvested plant tissues indicates similarities with shared microbes and differences with microbes unique to each plant or tissue type. The genera in common may indicate a similar source for the microbes, which may exist in the seeds (Nelson, 2018) or may be recruited from the environment. These microbes may have an impact on the plants’ health and success.

Examining the shared bacterial genera of the plant leaves indicated only six genera shared by all five crop types out of the 49 total genera identified. At least three of these genera are widely found in water (*Pseudomonas*, *Achromobacter*, and *Stenotrophomonas*). These microbes may be problematic in that they can be responsible for biofouling, but may also be antagonists to other bacteria or fungi, in particular to pathogens (Bonaterra et al., 2022). *Pseudomonas* and *Stenotrophomonas* have been detected in the ISS air and on station surfaces (Venkateswaran et al., 2014; Singh et al., 2018; Sielaff et al., 2019), introducing the possibility of transfer to the leaf surfaces as the fan on the Veggie unit draws in station air or when the bellows are lowered. Plant-associated *Pseudomonas* spp. are efficient colonizers of all sections of a plant. They may be deposited on the leaves and roots existing as saprophytes (Preston, 2004) and outcompete other microbes for plant exudates (Bonaterra et al., 2022). This competition has been shown to be effective against plant pathogens such as *Fusarium* and *Pythium* (Ellis et al., 1999; Duijff et al., 1994). The genus *Stenotrophomonas* comprises numerous species that have been detected on the ISS, including air, surfaces, water, and in previously grown Veggie crops (Venkateswaran et al., 2014; Khodadad et al., 2020; Hummerick et al., 2021). Some of these species can form favorable associations with plants, benefitting plant growth (Ryan et al., 2009). Other common genera included *Burkholderia* and *Sphingomonas*, both of which have been detected in the potable water system on the ISS and in the earlier days of shuttle (La Duc et al., 2003; Bruce et al., 2005; Ichijo et al., 2022).

The plant roots housed 79 genera in common across all plants that included the six genera common in the leaves. *Pseudomonas* was also highly abundant in the majority of plant root samples and has been detected in the ISS potable water supply (La Duc et al., 2003; Ichijo et al., 2022). Once introduced via the nutrient solution, there may be a potential for lateral and vertical transfer or by aerosolized solution as it is sprayed across the roots during the aeroponics nutrient delivery protocol. *Bacillus* spp. was also present in higher abundance in the plant root tissues. It was present in the root tissue and detected in the swab, increasing with each subsequent harvest and was the highest in wheat. *Bacillus* was also detected in very low abundance in the nutrient solution samples. Interestingly, in every wick sample, it was abundantly present, indicating that the bacteria may prefer, harbor, and grow not only on root tissues but also the wick materials, which were soaked by nutrient solutions during the XROOTS growouts and exposed to air. *Bacillus* species are normally found in the environment, such as in soil and water, and many are facultative anaerobes. Some *Bacillus* species can form endospores, which allow them to survive cleaning and may explain their presence throughout all harvests. Interestingly, *Bacillus* was lower in the tomato and pea plant tissues from the fourth harvest. Furthermore, *Bacillus* has been well studied as a potential biocontrol agent and has been shown to be effective against plant bacterial pathogens such as *Erwinia*, which was present in low numbers only in several wheat tissues and wicks. Further species-level identification is needed to understand more microbial migration behavior.

### 4.3 Common and unique microbial abundance among growouts and missions

A closer comparison of the XROOTS microbial communities with previous Veggie multi-crop demonstrations of leafy greens (VEG-03D, VEG-03E, and VEG-03F) on the ISS was conducted. The data indicated that the XROOTS crops had more unique bacterial genera than the common genera shared with previous Veggie tech demo crops in both leaf and root tissues (65 and 87 compared with 5 and 26, respectively). Several factors may have contributed to these findings. The Veggie tech demos were completed in 2017 and 2018, and numerous flight missions had been conducted before XROOTS, possibly introducing additional microbes to the ISS environment. There had also been many Veggie crop growouts before XROOTS. These growouts including VEG-03D, VEG-03E, and VEG-03F used sanitized seeds, ethylene oxide (ETO)-sanitized flight hardware, and sterilized substrates, therefore may not significantly introduce viable environmental microbes. On the other hand, plant growth environments may allow some enrichment and survival of plant-harboring or plant-associated microbes over time. Furthermore, all previous Veggie demonstrations were prepared with surface-sanitized seeds, which removed many of the bacteria and fungi prior to launch to the ISS. The seeds used in XROOTS were not surface-sanitized and may have increased the number of genera introduced to each XROOTS growout.

Interestingly, although many more unique microbes were identified in the XROOTS plant samples, the total counts of microbes detected in these plant samples were comparable to those in other Veggie growouts by culture-based analyses. This indicates that XROOTS aeroponic–hydroponic growouts may result in more diverse plant-associated microbiomes, which may have potential beneficial effects on plant health and microbiome balance. Many of the unique microorganisms (both bacteria and fungi) may serve as plant growth promoters, especially those in the rhizosphere. To make inferences on the plant–microbe interactions or the microbe–microbe interactions, additional studies would need to be conducted, especially as these interactions relate to the maintenance of plant health. This would be especially true for the controlled, closed environments on the ISS, as well as on lunar and Martian surfaces.

### 4.4 Microbial diversity, sources, and pathogen control

The bacterial and fungal communities that make up the plants’ microbiomes indicated differences in alpha diversity, with the numbers of species in the leaf tissue lower than those in the root tissue. The alpha diversity of different crop types also varied, ranging from 0.4 in radish to over 3 in wheat root tissue. This follows the same trends observed for the microbial count data, which had higher counts in the plant root tissues, a trend also generally seen in terrestrial agriculture and previous Veggie tech demos on the ISS (Khodadad et al., 2020; Hummerick et al., 2021). The microbial community of the phyllosphere is typically lower in diversity and microbial load due to lack of nutrients. In addition, the environmental factors on the ISS, such as higher CO_2_ levels and relatively low ambient relative humidity, provide a habitat unsuitable for high microbial growth (Bunchek et al., 2024). These would be common environmental residual effects that may influence the microbial communities (Daniels et al., 1985; Jin et al., 2022). Fungi presented the same trends as the bacterial community, with radish showing the lowest diversity and wheat the highest diversity.

As these plants were grown using an aeroponic–hydroponic method, some constituents of the microbial community could have originated from the potable water on the ISS, which is regularly monitored with culturable cell counts ranging from 0 to 10^4^ CFU (La Duc et al., 2003; Van Houdt et al., 2012; Ichijo et al., 2022). The dominant microbes identified in potable water included *Methylobacterium*, *Ralstonia*, *Sphingomonas*, and *Pseudomonas*, all of which were identified in both leaf and root tissues of the majority of the XROOTS plant samples (Van Houdt et al., 2012; Ichijo et al., 2022). Some species within these genera may be effective biocontrol agents or plant growth-promoting microbes (Bonaterra et al., 2022; Mehrabi et al., 2016; Sheridan et al., 2016).

Many plant pathogens are fungi, and two notable potential plant pathogens discovered in this study were *Fusarium*, an opportunistic pathogen, and *Blumeria*, an obligate biotrophic pathogen. Several species of *Fusarium* were identified, with the most abundant being *Fusarium oxysporum* and *F. solani*, both potential pathogens for tomato crops. Although present in the majority of plant samples, no visible indication of associated plant disease was detected (or observed). These same plant tissues also had increased abundance of *Pseudomonas* spp., which have been known to outcompete and reduce the numbers of *Fusarium* (Bonaterra et al., 2022; Mehrabi et al., 2016; Sheridan et al., 2016; Gaiero et al., 2013). Another fungus present in these plant tissues, *Trichoderma*, has been identified as a competitor to *Fusarium* and has the capability to inhibit its growth (Modrzewska et al., 2022). It is important to further identify these organisms to a species or strain taxon level as somespecies of *Fusarium* could be obligate pathogens to various other crops. A zinnia plant, part of a Veggie tech demo, displayed signs of fungal infection. Upon return to Earth, the zinnia leaf and root samples were analyzed, and the fungus was identified as *F. oxysporum* (Schuerger et al., 2021; Urbaniak et al., 2018). Analyses of every subsequent Veggie tech demo have identified *Fusarium* species as its presence has been established on the ISS.

*Blumeria graminis*, an Ascomycete fungus identified in many XROOTS plant samples, is known to be a phytopathogen that causes powdery mildew in a wide range of plants, including grasses and cereals (Inuma et al., 2007; Menardo et al., 2017). The infection can be identified by the appearance of white to grayish “splotches” or spots on plant leaf surfaces or stems. The signs of infection were observed on one of the wheat plants returned for microbial analysis, but the analysis also revealed the presence of *B. graminis* in tomato and pea plants without noticeable changes. As all three plants were intercropped during growout 3, it is possible that the fungi had the opportunity to spread to other plant tissues in proximity. All these findings highlight the importance of plant pathogen control including prevention, early detection, and mitigation.

## 5 Conclusion

The XROOTS was successfully tested on the ISS with three multi-crop growouts. As its aeroponic spray and the ebb and flow delivery system provided the nutrients to all the crops, some similarities were observed in the microbial communities between these harvests and the previous crops grown in the Veggie tech demos conducted in 2015–2018. There were a few shifts in the microbial composition in the root zone, and the appearance of multiple potential plant pathogens, along with many plant growth-promoting microbes and potential bacterial and fungal biocontrol agents, was identified. No pathogens related to food safety were detected.

An aeroponic-hydroponic system like XROOTS system may provide numerous advantages for growing edible crops on the ISS and on lunar or Martian surfaces as it potentially minimizes the amount of water required and lowers the upmass of transporting soil/substrates. However, there is a need for a better understanding of plant microbiome balance and plant–microbe interactions to maintain plant health in closed environments to enhance our knowledge for Earth-independent crop production operations and to enable the use of plant beneficial and biocontrol microorganisms to improve plant resilience and performance.

Resource Identification Initiative QIIME 2 View (RRID: SCR_018074)

DADA2 (RRID: SCR_023519)

ggplot2 (RRID: SCR_014601)

GraphPad Prism (RRID: SCR_002798)

Venny 2.1 (RRID: SCR_016561)

SILVA (RRID: SCR_006423)

R Project for Statistical Computing (RRID: SCR_001905)

tidyverse (RRID: SCR_019186)

phyloseq (RRID: SCR_013080)

kraken2 (RRID: SCR_026838)

UNITE (RRID: SCR_006518)

Pavian (RRID: SCR_016679)

## Data Availability

The original contributions presented in the study are publicly available. This data can be found here: NASA Open Science Data Repository, accession: OSD-773, doi: 10.26030/y0ng-8r17. This dataset is also available at NCBI BioProject, accession PRJNA1471327, https://www.ncbi.nlm.nih.gov/bioproject/?term=PRJNA1471327.
