# Supplementary material for: Microbial community characterization of multi-crop growouts in the XROOTS aeroponic–hydroponic system on the International Space Station
Source: Front Microbiomes. 2026 Jun 15;5:1779816. doi: 10.3389/frmbi.2026.1779816 (PMC13311008; doi:10.3389/frmbi.2026.1779816)
Supplement: Supplementary file 1 [file DataSheet1.docx]

Supplementary Figures 1 through 6


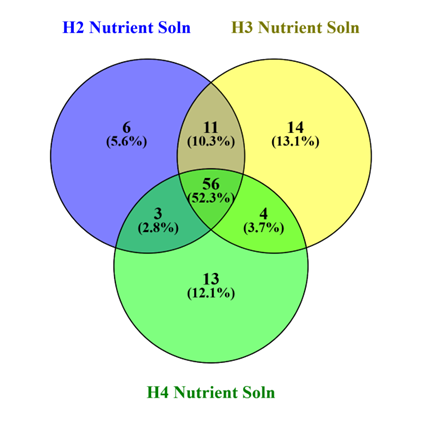


Supplementary Figure 1. Comparison of bacterial genera from the nutrient solution samples recovered from the 3 XROOTS growouts on ISS. *https://bioinfogp.cnb.csic.es/tools/venny/index.html*


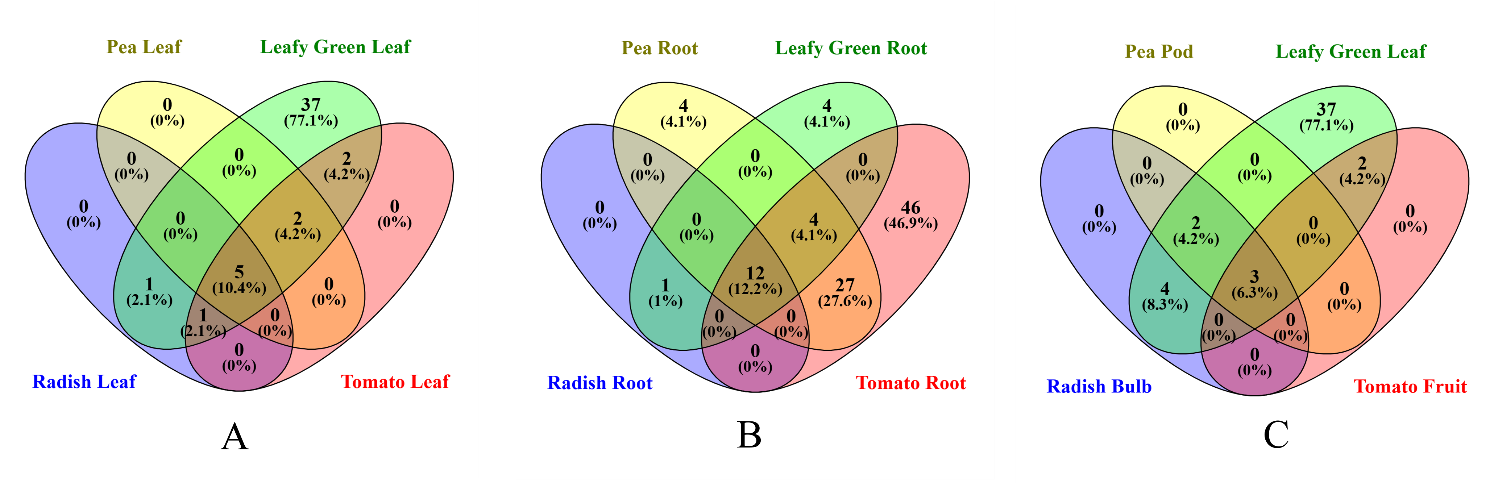


Supplementary Figure 2. Comparison fungi detected on plant leaf (A), plant root (B) and edible fruit (C) of different plants grown and harvested in XROOTS on ISS. The Venn diagrams were created using Venny 2.1. n≥1. *https://bioinfogp.cnb.csic.es/tools/venny/index.html*


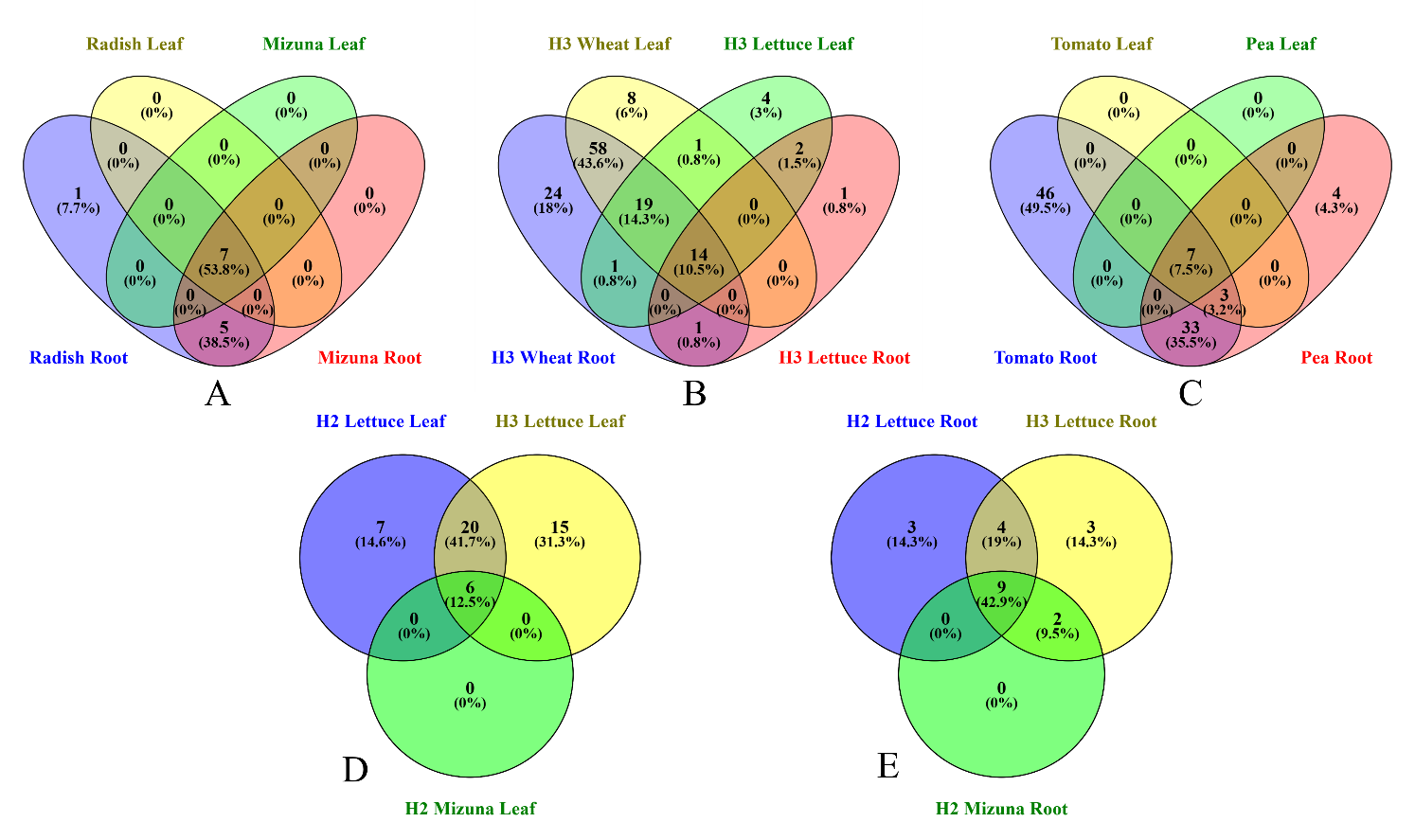


Supplementary Figure 3. Comparison of fungi of plant leaf and root tissue of plants grown during the same growouts on ISS in XROOTS root modules (A-C). Leafy greens leaf (D) and root (E) grown during harvests 2 and 3 are compared. The Venn diagrams were created using Venny 2.1. n≥1. *https://bioinfogp.cnb.csic.es/tools/venny/index.html*


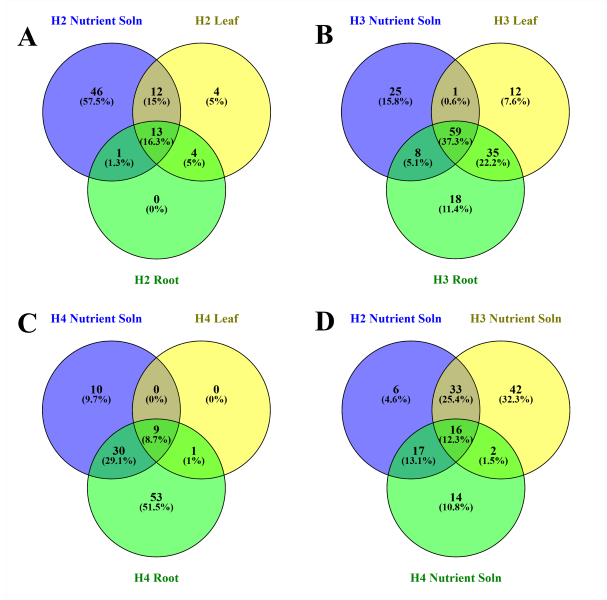


Supplementary Figure 4. Comparison of fungi identified in the nutrient solution samples taken from XROOTS on the ISS at different harvests. A-C compares plants and nutrient solution at the different harvests. Figure D compares the nutrient solution from the different harvest. The Venn diagrams were created using Venny 2.1. n≥1. *https://bioinfogp.cnb.csic.es/tools/venny/index.html*


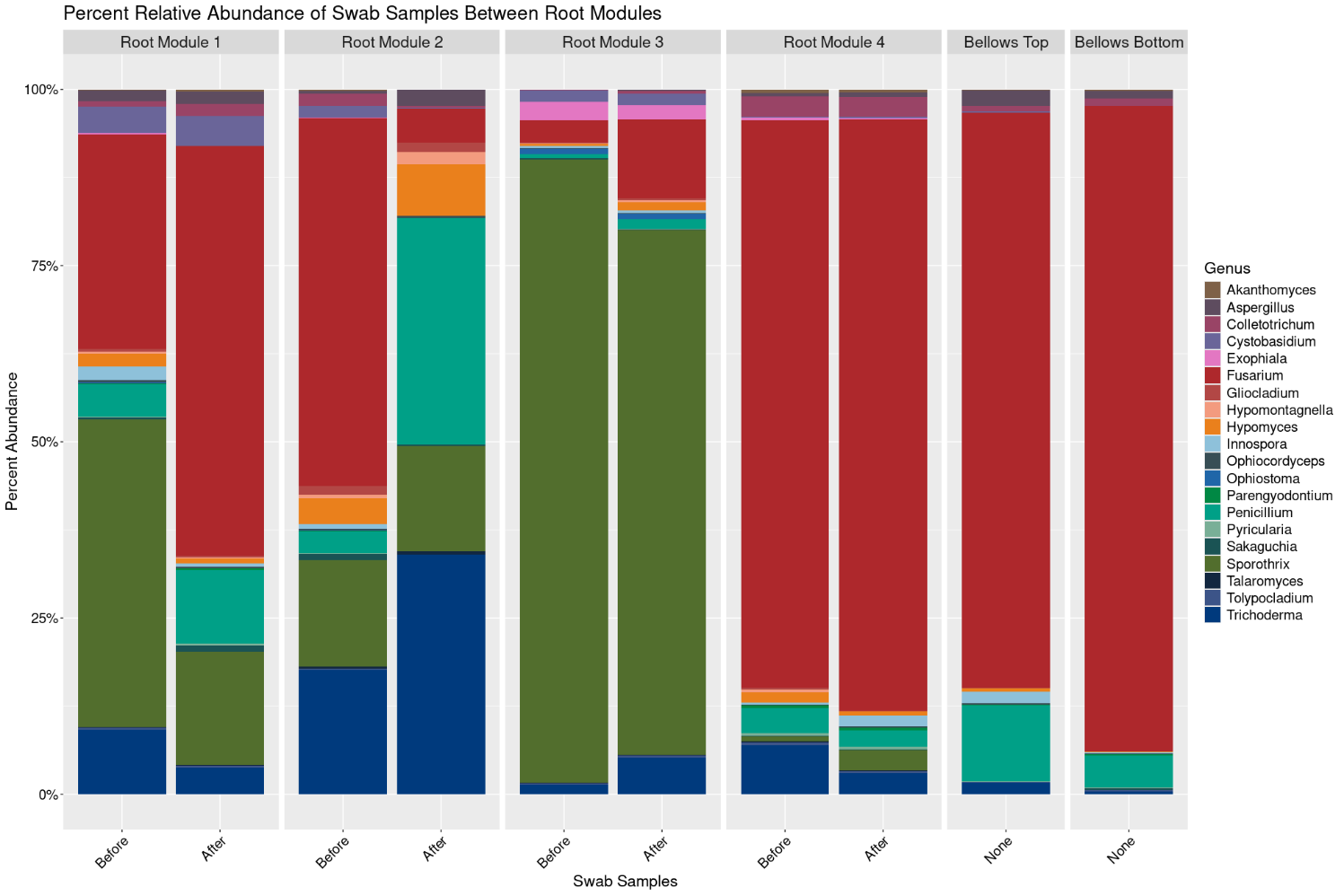
Supplementary Figure 5. Stacked bar plot of the top 20 fungal genera identified in the swab samples taken before and after the ProSan cleaning of the root modules. *Blumeria* was removed from this figure to better visualize the remaining fungi present in the samples.


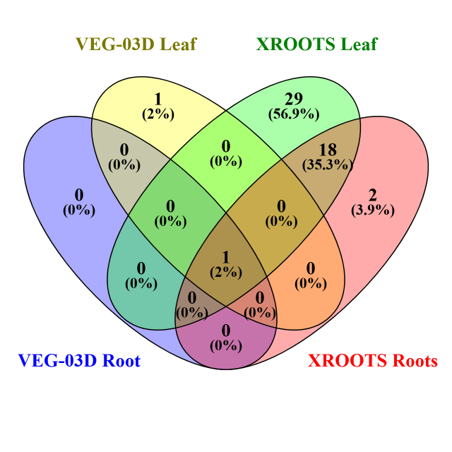


Supplementary Figure 6. A comparison of the fungi detected on leaf and root plant tissue from XROOTS and the VEG-03D technical demonstration. The Venn diagrams were created using Venny 2.1. n≥1. *https://bioinfogp.cnb.csic.es/tools/venny/index.html*
