# Supplementary material for: Microbial community characterization of multi-crop growouts in the XROOTS aeroponic–hydroponic system on the International Space Station
Source: Front Microbiomes. 2026 Jun 15;5:1779816. doi: 10.3389/frmbi.2026.1779816 (PMC13311008; doi:10.3389/frmbi.2026.1779816)
Supplement: Supplementary file 2 [file Table1.docx]

Supplementary Table 1. Mean CFU/g leaf and root tissue on TSA for aerobic bacteria and IMA for yeast and mold. * Indicates only one or two samples were available for analysis and not included in statistical analysis.

|  | CFU/g leaf | | CFU/g root | | CFU/g wick | |
| --- | --- | --- | --- | --- | --- | --- |
|  | TSA | IMA | TSA | IMA | TSA | IMA |
| Radish | 4.9 x 10^6^ | 4.0 x 10^4^ | 6.7 x 10^7^ | 6.2 x 10^5^ | 2.0 x 10^7^* | 7.0 x 10^5^* |
| Lettuce | 1.5 x 10^7^ | 9.6 x 10^5^ | 5.4 x 10^7^ | 2.1 x 10^6^ | 6.0 x 10^7^ * | 7.4 x 10^6^ |
| Wheat | 5.4 x 10^7^ | 6.8 x 10^5^ | 2.3 x 10^8^ | 8.0 x 10^5^ | 8.5 x 10^7^ | 3.1 x 10^6^ |
| Tomato | 1.0 x 10^7^ | 5.1 x 10^4^ | 4.9 x 10^8^* | 2.0 x 10^6^* | 2.9 x 10^8^* | 1.2 x 10^7^* |
| Mizuna mustard | 3.7 x 10^6^* | 2.5 x 10^4^* | 6.1 x 10^7^* | 1.2 x 10^4^* | No sample | No sample |
