# Supplementary material for: Microbial community characterization of multi-crop growouts in the XROOTS aeroponic–hydroponic system on the International Space Station
Source: Front Microbiomes. 2026 Jun 15;5:1779816. doi: 10.3389/frmbi.2026.1779816 (PMC13311008; doi:10.3389/frmbi.2026.1779816)
Supplement: Supplementary file 3 [file Table2.docx]

Supplementary Table 2. List of bacteria and fungi identified in each sample based upon isolate identification using Biolog or MicroSeq with the 16S rRNA gene or the D2 large ribosomal subunit on the ABI 3500 genetic analyzer. (Identified bacteria are in black font and fungi are in purple font). The + indicates the microbe is present and the – indicates its absence.

| **Radish-Harvest 2**  Identified isolates | Leaf top | Leaf Bottom | Root top | Root bottom | Bulb | Wick |
| --- | --- | --- | --- | --- | --- | --- |
| *Chryseobacterium taeanense* | **+** | **+** | **+** | **+** | **+** | **-** |
| *Mycobacterium setense* | **+** | **+** | **+** | **+** | **+** | **-** |
| *Pseudomonas putida* | **+** | **+** | **+** | **+** | **+** | **-** |
| *Micrococcus luteus D* | **+** | **+** | **+** | **+** | **+** | **-** |
| *Stenotrophomonas maltophilia* | **+** | **+** | **+** | **+** | **+** | **-** |
| *Bacillus tortis* | **-** | **+** | **-** | **+** | **-** | **-** |
| *Bacillus pseudomycoides/cereus* | **+** | **+** | **+** | **+** | **-** | **-** |
| *Paenibacillus forsythiae* | **-** | **-** | **-** | **+** | **-** | **-** |
| *Pseudomonas aeruginosa* | **-** | **+** | **+** | **+** | **-** | **-** |
| *Chryseobacterium gregarium* | **-** | **-** | **-** | **+** | **-** | **+** |
| *Bacillus pumilus/safensis* | **-** | **-** | **+** | **-** | **-** | **-** |
| *Fusarium sp* | **+** | **+** | **+** | **+** | **+** | **+** |
| *Rhodotorula mucilaginosa* | **+** | **+** | **+** | **+** | **+** | **+** |
| *Exophiala sp* | **+** | **+** | **+** | **+** | **+** | **-** |
| *Sporothix sp* | **+** | **+** | **+** | **+** | **-** | **+** |
| *Trichoderma sp* | **-** | **+** | **+** | **+** | **-** | **+** |

| **Mizuna-Harvest 2** | Leaf top | Leaf bottom | Root top | Root bottom |
| --- | --- | --- | --- | --- |
| *Chryseobacterium taeanense* | **+** | **+** | **+** | **+** |
| *Mycobacterium setense* | **+** | **+** | **+** | **+** |
| *Pseudomonas putida* | **+** | **+** | **+** | **+** |
| *Micrococcus luteus D* | **+** | **+** | **+** | **+** |
| *Stenotrophomonas maltophilia* | **+** | **+** | **+** | **+** |
| *Bacillus pseudomycoides/cereus* | **+** | **+** | **+** | **+** |
| *Pseudomonas aeruginosa* | **-** | **+** | **+** | **-** |
| *Fusarium sp* | **+** | **+** | **+** | **+** |
| *Rhodotorula mucilaginosa* | **-** | **+** | **+** | **+** |
| *Exophiala sp* | **-** | **-** | **+** | **-** |
| *Sporothix sp* | **-** | **-** | **+** | **-** |
| *Trichoderma sp* | **-** | **-** | **+** | **-** |

| **Outredgeous Lettuce-**  **Harvest 2** | Leaf top | Leaf bottom | Root top | Root bottom |
| --- | --- | --- | --- | --- |
| *Chryseobacterium taeanense* | **+** | **+** | **+** | **+** |
| *Mycobacterium setense* | **+** | **+** | **+** | **+** |
| *Pseudomonas putida* | **+** | **+** | **+** | **+** |
| *Micrococcus luteus D* | **+** | **+** | **+** | **+** |
| *Stenotrophomonas maltophilia* | **+** | **+** | **+** | **+** |
| *Bacillus tortis* | **-** | **-** | **+** | **-** |
| *Bacillus pseudomycoides/cereus* | **+** | **+** | **+** | **+** |
| *Pseudomonas aeruginosa* | **-** | **-** | **+** | **+** |
| *Microbacterium sp* | **-** | **-** | **-** | **+** |
| *Fusarium sp* | **+** | **+** | **+** | **+** |
| *Rhodotorula mucilaginosa* | **-** | **-** | **+** | **+** |
| *Sporothix sp* | **+** | **+** | **-** | **-** |
| *Trichoderma sp* | **-** | **-** | **-** | **+** |

| **Outredgeous lettuce-**  **Harvest 3** | Leaf top | Leaf bottom | Root top | Root bottom | Wick |
| --- | --- | --- | --- | --- | --- |
| *Chryseobacterium taeanense* | **+** | **+** | **+** | **+** | **+** |
| *Microbacterium maritypicum* | **+** | **+** | **+** | **+** | **+** |
| *Microbacterium species CDC A-4/A-5* | **+** | **+** | **+** | **+** | **+** |
| *Bacillus pumilus/safensis* | **+** | **-** | **+** | **+** | **-** |
| *Sporosarcina koreensis* | **+** | **+** | **+** | **+** | **+** |
| *Bacillus megaterium* | **+** | **+** | **+** | **-** | **+** |
| *Paenibacillus sanguinis* | **-** | **+** | **-** | **-** | **+** |
| *Bacillus sp* | **-** | **-** | **-** | **-** | **+** |
| *Lysinibacillus boronitolerans* | **+** | **+** | **+** | **-** | **+** |
| *Pseudomonas putida* | **-** | **-** | **+** | **+** | **+** |
| *Penicillium citrinum* | **+** | **+** | **+** | **+** | **+** |
| *Fusarium sp* | **+** | **+** | **+** | **+** | **+** |
| *Fusarium solani* | **+** | **+** | **+** | **+** | **+** |
| *Rhodotorula minuta* | **+** | **+** | **+** | **+** | **+** |
| *Trichoderma sp* | **+** | **-** | **+** | **+** | **+** |
| *Sporothix sp* | **-** | **-** | **-** | **+** | **+** |
| *Exophiala sp* | **-** | **-** | **-** | **+** | **-** |

| **Wheat-Harvest 3** | Leaf top | Leaf bottom | Root top | Root bottom | Wick |
| --- | --- | --- | --- | --- | --- |
| *Chryseobacterium taeanense* | **+** | **+** | **+** | **+** | **+** |
| *Microbacterium maritypicum* | **+** | **+** | **+** | **+** | **+** |
| *Microbacterium species CDC A-4/A-5* | **+** | **+** | **+** | **+** | **+** |
| *Bacillus pumilus/safensis* | **-** | **+** | **-** | **-** | **-** |
| *Sporosarcina koreensis* | **+** | **+** | **+** | **+** | **+** |
| *Bacillus megaterium* | **+** | **+** | **+** | **+** | **+** |
| *Paenibacillus sanguinis* | **+** | **+** | **-** | **-** | **-** |
| *Lysinibacillus boronitolerans* | **+** | **+** | **+** | **+** | **+** |
| *Pseudomonas putida* | **+** | **+** | **+** | **+** | **+** |
| *Pseudomonas aeruginosa* | **-** | **-** | **-** | **+** | **-** |
| *Penicillium citrinum* | **+** | **+** | **+** | **+** | **+** |
| *Fusarium sp* | **+** | **+** | **+** | **+** | **+** |
| *Fusarium solani* | **+** | **+** | **+** | **+** | **+** |
| *Rhodotorula minuta* | **+** | **-** | **+** | **-** | **+** |
| *Trichoderma sp* | **-** | **-** | **+** | **+** | **+** |
| *Sporothix sp* | **-** | **-** | **+** | **+** | **+** |
| *Exophiala sp* | **+** | **-** | **-** | **-** | **-** |
| *Algae-not defined* | **+** | **-** | **-** | **-** | **-** |

| **Tomato-Final harvest** | Leaves | Root top | Root bottom | Fruit | Wick |
| --- | --- | --- | --- | --- | --- |
| *Chryseobacterium taeanense* | **+** | **+** | **+** | **+** | **+** |
| *Rhizobium rhizogenes* | **-** | **+** | **+** | **+** | **+** |
| *Microbacterium esteraromaticum 97* | **-** | **+** | **+** | **+** | **+** |
| *Phyllobacterium sp* | **-** | **+** | **+** | **+** | **-** |
| *Stenotrophomonas maltophilia* | **-** | **-** | **+** | **+** | **+** |
| *Pseudomonas putida* | **+** | **+** | **+** | **-** | **+** |
| *Rhodococcus fascian B* | **-** | **+** | **+** | **-** | **+** |
| *Micrococcus luteus D* | **-** | **+** | **-** | **+** | **+** |
| *Bacillus pumilus/safensis* | **+** | **-** | **-** | **+** | **+** |
| *Pseudomonas aeruginosa* | **-** | **-** | **-** | **+** | **+** |
| *Penicillium sp* | **+** | **+** | **-** | **+** | **+** |
| *Fusarium sp* | **+** | **+** | **+** | **+** | **+** |
| *Exophiala sp* | **-** | **-** | **-** | **-** | **+** |
| *Sporothrix sp* | **+** | **-** | **-** | **-** | **+** |
| *Fusarium solani* | **-** | **-** | **-** | **+** | **+** |
| *Rhodotorula mucilaginosa* | **-** | **+** | **-** | **+** | **-** |
| *Trichoderma sp* | **-** | **+** | **+** | **+** | **-** |

| **Pea-Final harvest** | Leaves | Root top | Root bottom | Pod | Wick |
| --- | --- | --- | --- | --- | --- |
| *Chryseobacterium taeanense* | **-** | **+** | **+** | **+** | **+** |
| *Rhizobium rhizogenes* | **+** | **+** | **+** | **+** | **+** |
| *Microbacterium esteraromaticum 97* | **+** | **+** | **+** | **+** | **+** |
| *Stenotrophomonas maltophilia* | **+** | **+** | **+** | **+** | **+** |
| *Pseudomonas putida* | **+** | **+** | **+** | **-** | **+** |
| *Pelomonas saccharophila* | **+** | **+** | **+** | **+** | **+** |
| *Rhodococcus fascian B* | **-** | **+** | **+** | **+** | **+** |
| *Micrococcus luteus D* | **+** | **+** | **+** | **-** | **+** |
| *Bacillus pumilus/safensis* | **-** | **-** | **-** | **+** | **-** |
| *Pseudomonas aeruginosa* | **-** | **-** | **-** | **+** | **+** |
| *Bacillus sp* | **-** | **+** | **-** | **-** | **-** |
| *Penicillium sp* | **+** | **+** | **-** | **+** | **+** |
| *Fusarium sp* | **+** | **+** | **+** | **+** | **+** |
| *Exophiala sp* | **-** | **-** | **+** | **-** | **+** |
| *Sporothix sp* | **-** | **-** | **+** | **-** | **+** |
| *Fusarium solani* | **-** | **+** | **+** | **-** | **-** |

| **Water-All harvests** | Harvest 2 | Harvest 3 | Harvest 3 | Harvest 4 | |
| --- | --- | --- | --- | --- | --- |
| *Chryseobacterium taeanense* | **+** | **-** | **-** | | **-** |
| *Stenotrophomonas maltophilia* | **+** | **-** | **-** | | **+** |
| *Bacillus pumilus/safensis* | **+** | **-** | **+** | | **+** |
| *Pseudomonas aeruginosa* | **+** | **-** | **-** | | **-** |
| *Microbacterium maritypicum* | **-** | **+** | **-** | | **-** |
| *Microbacterium species CDCA-4/CDCA-5* | **-** | **+** | **-** | | **-** |
| *Sporosarcina koreensis* | **-** | **+** | **+** | | **-** |
| *Bacillus megaterium* | **-** | **+** | **-** | | **-** |
| *Bacillus sp* | **-** | **+** | **-** | | **+** |
| *Rhizobium rhizogenes* | **-** | **-** | **-** | | **+** |
| *Microbacterium esteraromaticum 97* | **-** | **-** | **-** | | **+** |
| *Pseudomonas putida* | **-** | **-** | **-** | | **+** |
| *Sporothix sp* | **+** | **+** | **+** | | **+** |
| *Fusarium sp* | **-** | **+** | **-** | | **-** |
| *Fusarium solani* | **-** | **+** | **-** | | **-** |
| *Trichoderma sp* | **-** | **+** | **-** | | **-** |
| *Exophiala sp* | **-** | **+** | **+** | | **-** |
| *Rhodotorula mucilaginosa* | **-** | **-** | **-** | | **+** |

| **Swabs-Harvest 2** | RM 1 before sanitization | RM1 after sanitization | RM3 before sanitization | RM3 after sanitization |
| --- | --- | --- | --- | --- |
| *Chryseobacterium taeanense* | + | + | + | + |
| *Mycobacterium setense* | + | + | + | + |
| *Pseudomonas putida* | + | + | + | + |
| *Micrococcus luteus D* | + | + | + | + |
| *Stenotrophomonas maltophilia* | + | + | + | + |
| *Bacillus tortis* | + | - | - | - |
| *Bacillus pseudomycoides/cereus* | + | + | + | + |
| *Paenibacillus forsythiae* | - | + | - | + |
| *Chryseobacterium gregarium* | - | - | + | - |
| Bacillus pumilus/safensis | - | + | - | + |
| *Fusarium sp* | **+** | **+** | **+** | **+** |
| *Rhodotorula mucilaginosa* | **-** | **-** | **-** | **+** |
| *Exophiala sp* | **-** | **-** | **+** | **+** |
| *Sporothix sp* | **+** | **+** | **-** | **+** |
| *Trichoderma sp* | **+** | **-** | **+** | **+** |

| **Swabs- Harvest 3** | RM 1 before sanitization | RM1 after sanitization | RM2 before sanitization | RM2 after sanitization |
| --- | --- | --- | --- | --- |
| *Chryseobacterium taeanense* | **+** | **+** | **+** | **+** |
| *Microbacterium maritypicum* | **+** | **+** | **+** | **+** |
| *Microbacterium species CDC A-4/A-5* | **+** | **+** | **+** | **+** |
| *Bacillus pumilus/safensis* | **+** | **+** | **-** | **-** |
| *Sporosarcina koreensis* | **+** | **+** | **+** | **-** |
| *Bacillus megaterium* | **+** | **+** | **+** | **-** |
| *Paenibacillus sanguinis* | **-** | **+** | **-** | **-** |
| *Bacillus sp* | **-** | **+** | **-** | **-** |
| *Penicillium citrinum* | **+** | **+** | **+** | **+** |
| *Fusarium sp* | **+** | **+** | **+** | **+** |
| *Fusarium solani* | **+** | **+** | **+** | **+** |
| *Rhodotorula minuta* | **-** | **+** | **+** | **-** |
| *Trichoderma sp* | **-** | **-** | **+** | **+** |
| *Sporothix sp* | **+** | **+** | **+** | **+** |
| *Exophiala sp* | **-** | **+** | **+** | **+** |
| Algae-not defined | **-** | **-** | **+** | **-** |

| **Swabs-Final harvest** | Bellows  Near bottom | Bellows  Near top | RM4 before sanitization | RM4 after sanitization |
| --- | --- | --- | --- | --- |
| *Chryseobacterium taeanense* | **+** | **-** | **-** | **-** |
| *Rhizobium rhizogenes* | **+** | **+** | **-** | **-** |
| *Microbacterium esteraromaticum 97* | **+** | **+** | **-** | **-** |
| *Pseudomonas aeruginosa* | **+** | **-** | **-** | **-** |
| *Bacillus sp* | **+** | **+** | **-** | **+** |
| *Penicillium sp* | **+** | **+** | **+** | **+** |
| *Fusarium sp* | **-** | **+** | **+** | **+** |
| *Exophiala sp* | **-** | **-** | **+** | **+** |
| *Sporothrix sp* | **-** | **-** | **+** | **+** |
| *Trichoderma sp* | **-** | **-** | **+** | **+** |
