# Supplementary material for: Microbial community characterization of multi-crop growouts in the XROOTS aeroponic–hydroponic system on the International Space Station
Source: Front Microbiomes. 2026 Jun 15;5:1779816. doi: 10.3389/frmbi.2026.1779816 (PMC13311008; doi:10.3389/frmbi.2026.1779816)
Supplement: Supplementary file 4 [file Table3.docx]

Supplementary Table 3. Alpha diversity as determined by the Shannon Index and the number of species identified in each 16S bacterial community. The community was determined with the V4 region of the 16S rRNA gene and identified using SILVA v 138.99 and QIIME V2.
