# Supplementary material for: Microbial community characterization of multi-crop growouts in the XROOTS aeroponic–hydroponic system on the International Space Station
Source: Front Microbiomes. 2026 Jun 15;5:1779816. doi: 10.3389/frmbi.2026.1779816 (PMC13311008; doi:10.3389/frmbi.2026.1779816)
Supplement: Supplementary file 5 [file Table4.docx]

Supplementary Table 4. Alpha diversity as determined by the Shannon Index and the number of species identified in each ITS fungal community. Identification was completed using KRAKEN and UNITE database.
