# Supplementary material for: Microbial community characterization of multi-crop growouts in the XROOTS aeroponic–hydroponic system on the International Space Station
Source: Front Microbiomes. 2026 Jun 15;5:1779816. doi: 10.3389/frmbi.2026.1779816 (PMC13311008; doi:10.3389/frmbi.2026.1779816)
Supplement: Supplementary file 6 [file Table5.docx]

Supplementary Table 5. Adonis analysis results for radish and wheat grown in the XROOTS aeroponic-hydroponic system on the International Space Station. These adonis results are in support of the Bray-Curtis Beta Diversity analysis illustrated in the PCoA.

| **Adonis Results** | |  |  |  |  |  |
| --- | --- | --- | --- | --- | --- | --- |
| **Radish** | **Df** | **SumsOfSqs** | **MeanSqs** | **F.Model** | **R2** | **Pr(>F)** |
| **Sample Type** | 5 | 2.3473 | 0.4694 | 1.9035 | 0.5139 | 0.014 |
| **Residuals** | 9 | 2.2196 | 0.2466 | NA | 0.4860 | NA |
| **Total** | 14 | 4.5669 | NA | NA | 1 | NA |
|  |  |  |  |  |  |  |
| **Wheat** |  |  |  |  |  |  |
| **Sample Type** | 4 | 1.845 | 0.4614 | 3.2686 | 0.5666 | 0.001 |
| **Residuals** | 10 | 1.4118 | 0.1411 | NA | 0.4333 | NA |
| **Total** | 14 | 3.2577 | NA | NA | 1 | NA |
